# Biomolecular condensation orchestrates clathrin-mediated endocytosis in plants

**DOI:** 10.1038/s41556-024-01354-6

**Published:** 2024-02-12

**Authors:** Jonathan Michael Dragwidge, Yanning Wang, Lysiane Brocard, Andreas De Meyer, Roman Hudeček, Dominique Eeckhout, Peter Grones, Matthieu Buridan, Clément Chambaud, Přemysl Pejchar, Martin Potocký, Joanna Winkler, Michaël Vandorpe, Nelson Serre, Matyáš Fendrych, Amelie Bernard, Geert De Jaeger, Roman Pleskot, Xiaofeng Fang, Daniël Van Damme

**Affiliations:** 1 Department of Plant Biotechnology and Bioinformatics, Ghent University, Technologiepark 71, 9052 Ghent, Belgium; 2 VIB Center for Plant Systems Biology, Technologiepark 71, 9052 Ghent, Belgium; 3 Center for Plant Biology, School of Life Sciences, Tsinghua University, Beijing, China; 4 Univ. Bordeaux, CNRS, INSERM, Bordeaux Imaging Center, BIC, UAR 3420, US 4, F-33000 Bordeaux, France; 5 Institute of Experimental Botany of the Czech Academy of Sciences, Rozvojová 263, 16502 Prague 6, Czech Republic; 6 Laboratoire de Biogenèse Membranaire, UMR 5200, CNRS, Univ. Bordeaux, F-33140 Villenave d’Ornon; 7 Department of Experimental Plant Biology, Faculty of Sciences, Charles University, Prague, Czech Republic

## Abstract

Clathrin-mediated endocytosis is an essential cellular internalisation pathway involving the dynamic assembly of clathrin and accessory proteins to form membrane-bound vesicles. The evolutionarily ancient TSET/TPLATE complex (TPC) plays an essential, but not well-defined role in endocytosis in plants. Here, we show that two highly disordered TPC subunits, AtEH1 and AtEH2 function as scaffolds to drive biomolecular condensation of the complex. These condensates specifically nucleate on the plasma membrane through interactions with anionic phospholipids, and facilitate the dynamic recruitment and assembly of clathrin, early-, and late-stage endocytic accessory proteins. Importantly, condensation promotes ordered clathrin assemblies. TPC-driven biomolecular condensation thereby facilitates dynamic protein assemblies throughout clathrin-mediated endocytosis. Furthermore, we show that a disordered region of AtEH1 controls the material properties of endocytic condensates *in vivo*. Alteration of these material properties disturbs the recruitment of accessory proteins, influences endocytosis dynamics, and impairs plant responsiveness. Our findings reveal how collective interactions shape endocytosis.

## Introduction

Clathrin-mediated endocytosis (CME) is an essential internalisation pathway necessary for many cellular processes, including signalling, immune responses, and nutrient uptake^1,2^. Endocytosis is initiated through a network of transient interactions between cargo, adaptor proteins, membrane lipids, and the coat protein clathrin on the plasma membrane. Following initiation, clathrin polymerises into a lattice which bends as the membrane invaginates, leading to dynamin-dependent scission and release of the formed clathrin-coated vesicle (CCV)^3^. The assembly of these clathrin lattices is highly dynamic, and depends on a network of early accessory proteins which interact with the adaptor protein complex AP-2 to stabilise clathrin-coated pits^4,5^. Core endocytic machinery was present in the last eukaryotic common ancestor^6,7^, suggesting that the underlying principles that facilitate CCV assembly during endocytosis are largely conserved.

One key evolutionary difference is that amoeba and plants retained an ancient endocytic complex termed TSET, or TPLATE complex (TPC), which was lost from metazoan and fungal lineages^8,9^. TPC is essential for plant life, as viable knock-out mutants in individual subunits have not been identified^9–11^. Recent studies have established the critical role of TPC during endocytosis by demonstrating its interaction with many essential endocytic components, including anionic phospholipids^12,13^, AP-2^9,14^, clathrin^15^, and ubiquitinated cargo^16^. Furthermore, destabilisation of TPC leads to impaired endocytic internalisation, and is correlated with a disruption in membrane bending during clathrin-coated pit formation^17,18^. TPC is present throughout endocytosis progression, from the earliest detectable phase until vesicle scission and uncoating^9,19^. Collectively, these studies support a multi-functional role for TPC during endocytosis.

While TPC shares structural homology with COPI and AP-complexes^7,13^, its subunits do not have direct orthologs to metazoan or fungal proteins. However, TPC subunits contain common vesicle trafficking related domains and motifs which has provided insight into their molecular function^20^. Notably, the plant specific TPC subunits AtEH1/Pan1 (AtEH1) and AtEH2/Pan1 (AtEH2) share partial homology to the Eps15 homology (EH) domains proteins Eps15 and Intersectin in humans, and Ede1p and Pan1p in yeast. A conserved function in autophagy of AtEH proteins^11^, and Ede1p and Pan1p in yeast^21,22^ support the idea that they are functional homologs. We previously showed that AtEH1 co-purifies with AP-2^9,14^, and that AtEH1 interacts with anionic phospholipids and cargo^12^. Interactomics and live cell imaging suggest TPC assembles as an octameric complex on the membrane during the initiation of endocytosis^9,23^, with the lipid binding proteins AtEH1, AtEH2, and the muniscin-like protein TML forming an interface with the plasma membrane^13^. In comparison, human Eps15, intersectin and the muniscin FCHo mediate multivalent interactions between themselves, anionic phospholipids^24,25^, and AP-2^26–28^ to promote the initiation and growth of clathrin coated pits during endocytosis^29–31^.

Recently, biomolecular condensates, which can be assembled through phase separation has emerged as a fundamental mechanism to compartmentalise cellular functions^32,33^. In animals and yeast, Eps15/FCHo and Ede1 have been shown to promote the formation of condensates during the initiation of endocytosis^25,34^. Here, we demonstrate that in plants AtEH proteins drive condensation of TPC and the recruitment of accessory proteins throughout endocytosis, from initiation to scission. Using CLEM-ET, we found that clathrin forms organised lattice-like assemblies within these condensates. We show that phospholipid binding via the EH domains of AtEH1 drive condensate nucleation, while the sequence chemistry of the intrinsically disordered region 1 (IDR1) regulates condensate material properties. Moreover, altering these properties through manipulation of the IDR interaction strength influences endocytic dynamics and the responsiveness of plants.

## Results

### AtEH1 and AtEH2 phase separate *in vivo* and *in vitro*

**Extended Data Fig. 1 (Related to Figure 1):**
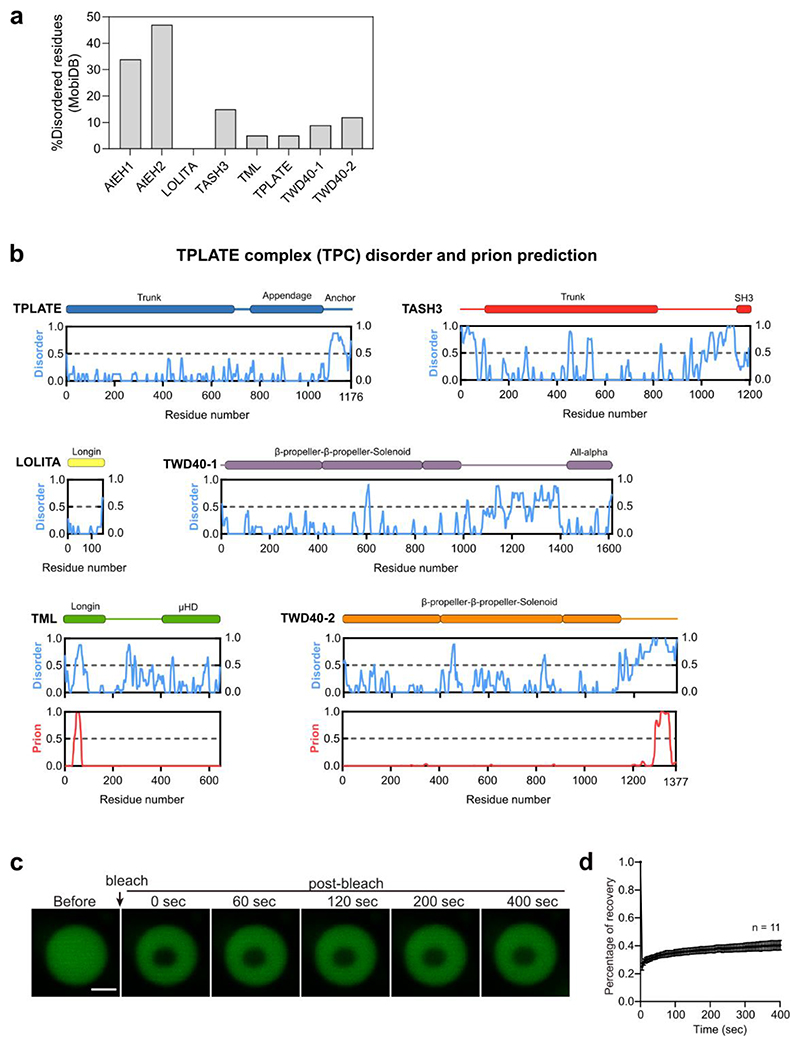
TPC disorder prediction, and AtEH1 *in vitro* FRAP assay **a**, Plot of the proportion of disordered residues for TPC subunits. **b**, Plot of TPC subunits showing prediction of disordered (MobiDB, blue), and prion-like (PLAAC, red) residues. Regions with values >0.5 are considered disordered, or prion-like respectively. Prion-like residues were only identified in TML and TWD40-2 subunits. (**c,d**) *In vitro* FRAP assay of purified GFP-AtEH1_FL_ protein. Data is mean ± SD. Scale bar = 2 μm. The experiment in **c** was performed two times with similar results.. Source data are provided as a Source Data file.

**Figure 1:**
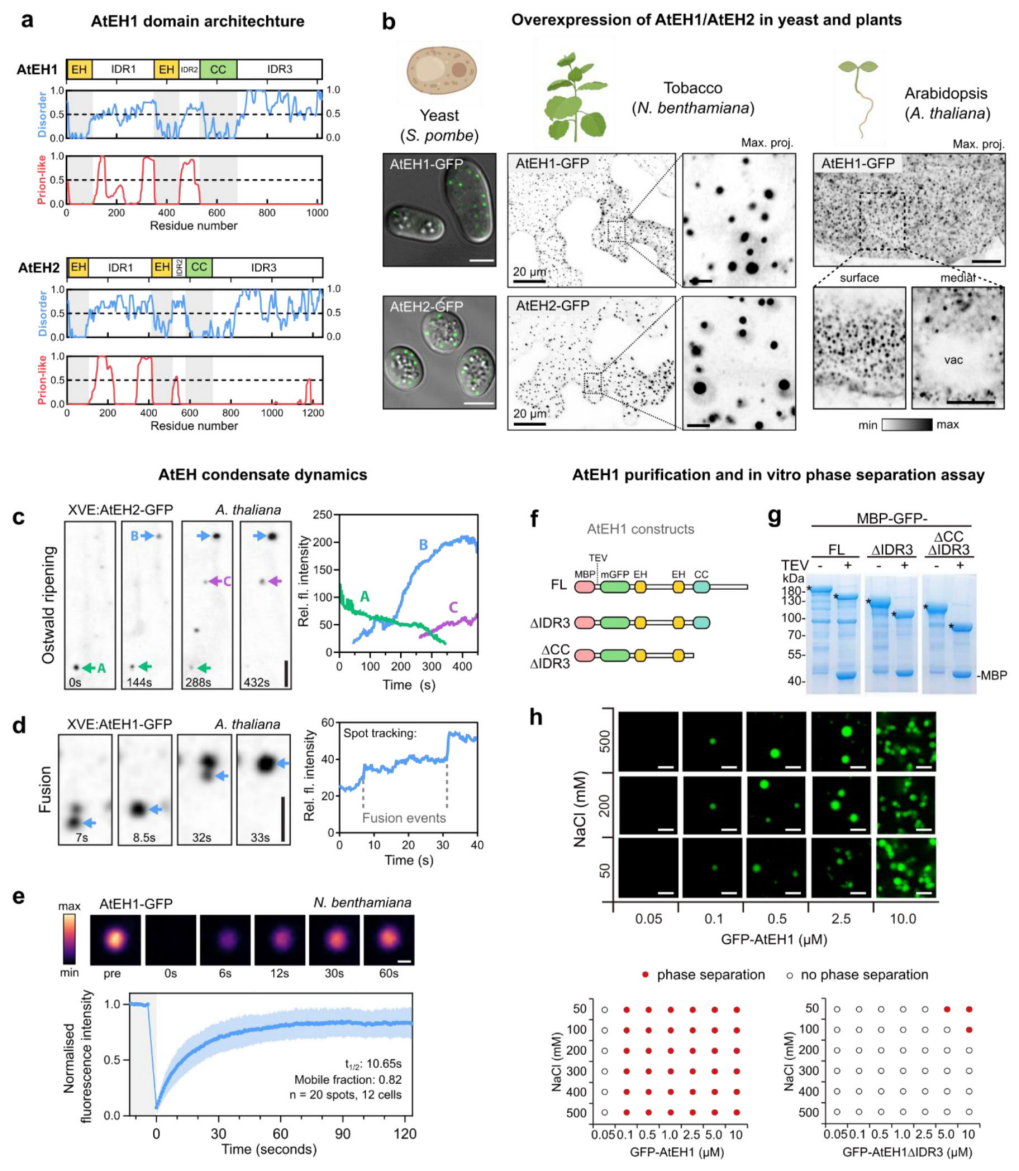
AtEH proteins phase separate *in vivo* and *in vitro* **a**, AtEH1 and AtEH2 domain architecture and prediction of disordered (MobiDB consensus) and prion-like (PLAAC) residues. Regions with values > 0.5 are considered disordered or prion-like, respectively. EH, Eps15 homology; CC, coiled-coil. **b**, Representative Airyscan images of AtEH1-GFP and AtEH2-GFP overexpressed in yeast (*S. pombe*), *N. benthamiana* epidermal cells (UBQ10:AtEH-mGFP), and in stable *A. thaliana* root epidermal cells (35S:AtEH1-GFP). (**c**-**d**) Time-lapse imaging of AtEH1 and AtEH2 under control of a β-estradiol inducible promoter (XVE) after 38 (**c**) or 22 (**d**) hours induction in *A. thaliana* root epidermal cells. Spot tracking and quantification of fluorescence intensity reveals puncta growth and shrinking through Ostwald ripening (**c**), and puncta fusion (**d**). **e**, Fluorescence recovery after photobleaching (FRAP) of AtEH1-GFP condensates in *N. benthamiana* epidermal cells. Data is mean ± SD. (**f**-**h**) Schematic of AtEH1 constructs used for recombinant protein purification (**f**). Constructs were purified as GFP-fusion proteins using an N-terminally located Maltose Binding Protein (MBP) tag with a Tobacco Etch Virus (TEV) cleavage site. **g**, Coomassie-stained SDS-PAGE gel of purified AtEH1 protein before and after TEV cleavage; * indicates AtEH1. **h**, *in vitro* phase separation assay and phase diagram of recombinant GFP-AtEH1. GFP-AtEH1_ΔCCΔIDR3_ did not phase separate at the tested conditions. Scale bars = 5 μm (**b, c, d**), 1 μm (**e**), 2 μm (**h**), or as otherwise indicated. For **b-e, g-h** experiments were performed three, or two times, respectively with similar results. **See also** Extended Data Fig. 1. Source data are provided as a Source Data file.

Sequence analysis revealed that AtEH1 and AtEH2 are highly disordered compared to other TPC subunits (Extended Data Fig. 1a,b), containing IDRs and prion-like domains (PrLD) (Fig. 1a), common features of proteins that phase separate^35^. AtEH proteins formed cytosolic and membrane associated puncta when overexpressed transiently in tobacco (*Nicotiana benthamiana*), in stable *Arabidopsis thaliana* lines, and in yeast (*Schizosaccharomyces pombe*) (Fig. 1b), consistent with previous studies^11^. These punctate compartments showed features typical of phase separated droplets, including dynamic growth and shrinking consistent with Ostwald ripening (Fig. 1c), fusion (Fig. 1d), and rapid protein exchange with the cytosolic pool (Fig. 1e).

Since AtEH1 and AtEH2 have a similar structure and ability to phase separate, we focused on AtEH1 due to its more central location in TPC compared to AtEH2^13^. We next performed *in vitro* phase separation assays using recombinantly purified full length AtEH1 (AtEH1_FL_), an IDR3 truncation (AtEH1_ΔIDR3_), and a coiled-coil (CC) and IDR3 truncation (AtEH1_ΔCCΔIDR3_) (Fig. 1f and g). AtEH1_FL_ phase separated at relatively low protein concentrations (≤ 0.1 µM), but showed limited fluorescence recovery (Fig. 1h and Extended Data Fig. 1c), suggesting that intra-molecular interactions promote phase separation but limit molecular re-arrangement in the absence of other components. Truncation of the IDR3, or both IDR3 and CC domains reduced, or abolished phase separation respectively (Fig. 1h), suggesting these domains promote phase separation *in vitro*.

### AtEH1 IDRs and structured domains promote condensation

**Extended Data Fig. 2 (Related to Figure 2):**
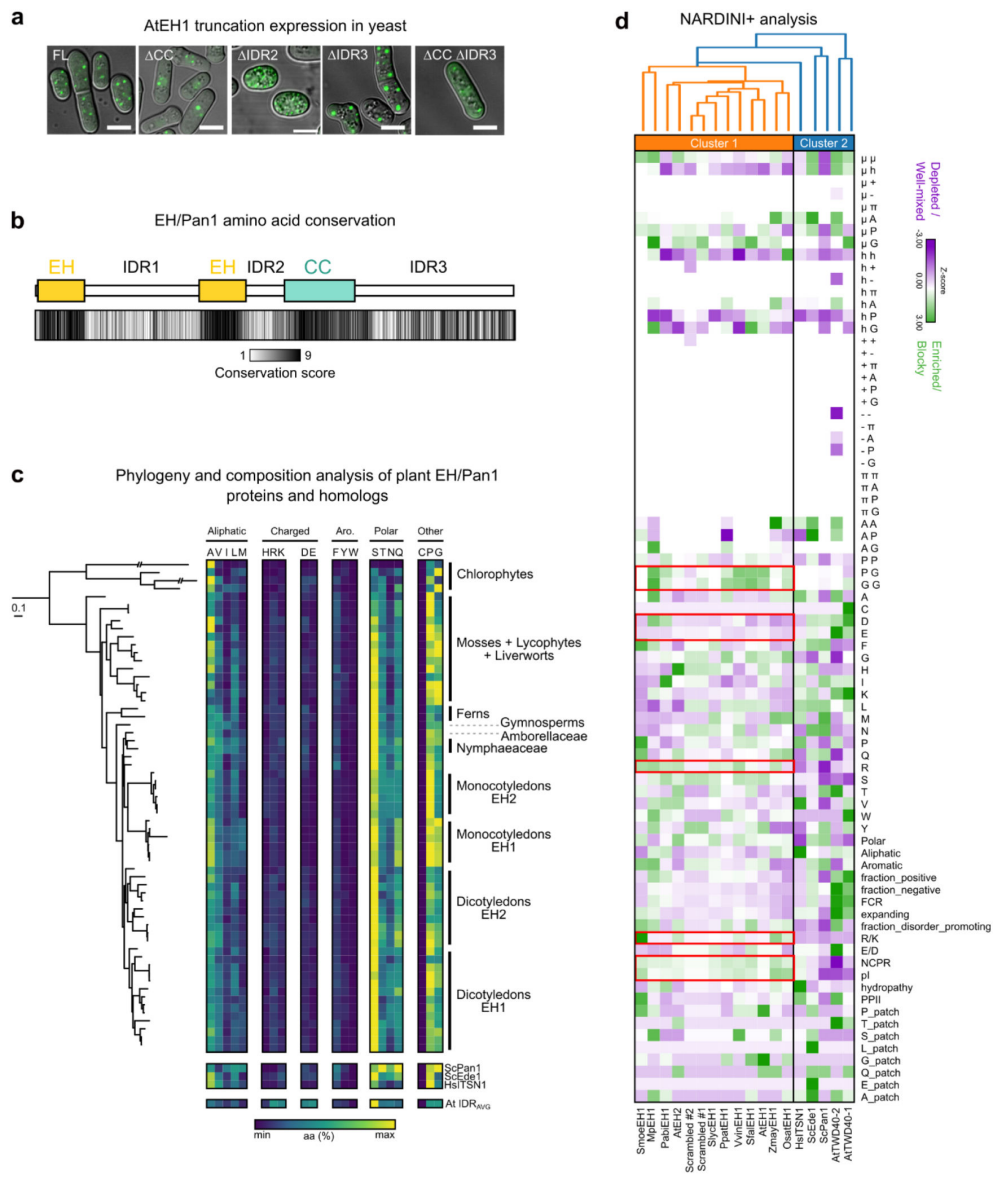
AtEH1 truncation construct expression in yeast, and evolutionary comparison of EH/Pan1 IDR1 amino acid composition across Archaeplastida **a**, Expression and condensate formation capacities of AtEH1 domain constructs in *S. pombe*. Scale bars = 5 μm. **b**, Conservation of amino acids at the single residue level based on Consurf analysis calculated using 128 AtEH homologous sequences throughout plant evolution. The average conservation score is indicated for each region (1 = 0% conservation, 9 = 100% conservation). IDR1 is highly variable at the individual amino acid level. **c**, Phylogenetic tree representing the maximum likelihood phylogeny of selected EH proteins. The phylogenetic tree was arbitrarily rooted to reflect phylogenetic relationships between chlorophyte and streptophyte lineages. The scale bar represents 0.1 amino acid substitution per site. The composition of the IDR1 for EH/Pan1 proteins is shown, with each amino acid plotted with the total number of each residue indicated. EH/Pan1 homologs from yeast (ScPan1, ScEde1), human (ITSN1), and the *A. thaliana* proteome average IDR are also indicated. **d**, NARDINI+ analysis of IDR patterning and compositional features from the IDR1 of AtEH1, AtEH2, and scrambled AtEH1 variants, homologous IDRs from liverwort (MpEH1), yeast (ScPan1, ScEde1), and human (HsITSN1) (used in Fig. 2c), IDRs from the TPLATE complex subunits TWD40-1/2, and the equivalent IDR from selected AtEH homologs throughout the plant kingdom. Z-score matrices are shown, with positive z-scores indicating non-random segregation between two types of residues, a blocky distribution of one type of residue, or the enrichment of the given sequence feature. Negative z-scores indicate non-random mixing between two types of residues, a uniform distribution of one type of residue, or the depletion of the given sequence feature. Red boxes indicate notable compositional features that differ between the two clusters including charged residue sequence features. Z-scores ≤ -1.5 and ≥ 1.5 are considered significant. μ, polar; h, hydrophobic; +, basic; –, acidic; π, aromatic; pI, isoelectric point; NCPR, net charge per residue; PPII, polyproline II propensity. The sequences used to construct the alignment in panel **b** and the phylogenetic tree in panel **c** are provided in the Source Data file. The experiment in **a** was performed two times with similar results.

**Figure 2:**
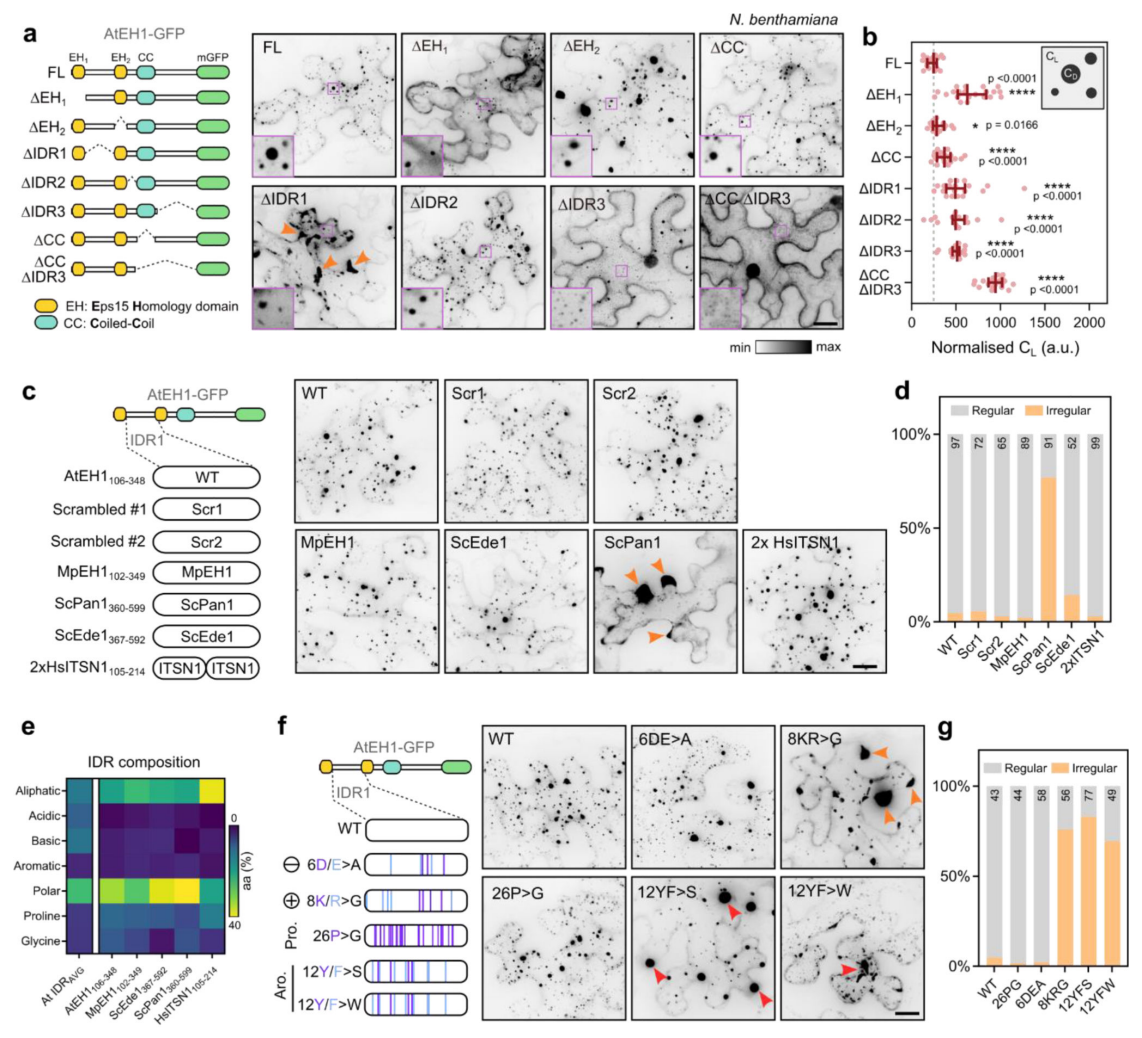
Condensation of AtEH1 is controlled by multiple regions, including IDR1 **a**, Schematic of AtEH1 truncation constructs (UBQ10:AtEH1_domain_-GFP) and representative image in *N. benthamiana*. Insets show zoom in of condensates (dense phase; C_D_) and cytosol (light phase; C_L_). **b**, Quantification of cytosolic protein concentration (light phase; C_L_). Bars indicate median ± 95% CI. Higher values indicate a reduced ability to phase separate. Statistics indicate significance to FL; *p < 0.05, ****p <0.0001, unpaired t-test with Welch’s correction; n = 18, 21, 15, 19, 17, 17, 19, 19 cells. **c**, IDR swap experiment. The IDR1 of AtEH1 was scrambled (Scr) or replaced with an equivalent IDR from AtEH1 homologs from liverwort (*Marchantia polymorpha*; MpEH1), yeast (*Saccharomyces cerevisiae*; ScPan1, ScEde1), and human (*Homo sapiens*; HsITSN1). Representative images in *N. benthamiana* are shown. Orange arrowheads indicate irregular condensates appearing in cell lobes. **d**, Quantification of the condensate distribution in cells. The number of cells analysed is indicated. **e**, Heat map of amino acid composition of IDRs from the *A. thaliana* IDR proteome, and from the IDR1 of AtEH1 and equivalent sequences from related proteins. AtEH1 homologs are more similar to each other than to the average *A.thaliana* IDR sequence. Notably, ScPan1 contains few basic residues. (**f**-**g**) AtEH1 IDR1 mutation experiment. Proline, basic, acidic, and aromatic residues of the IDR1 of AtEH1 were mutated. Representative images in *N. benthamiana* are shown. Orange arrowheads indicate irregular condensates appearing at cell lobes, red arrowheads indicate condensates with abnormal morphology. **g**, Quantification of condensate distribution in cells. The number of cells analysed is indicated. Scale bars = 20 μm. For **a-d,f-g** experiments were performed three times with similar results. **See also** Extended Data Fig. 2. Source data are provided as a Source Data file.

We next asked whether we could determine the region(s) which promote condensation of AtEH1 *in planta*. To test this, we systematically removed each IDR and structured domain in AtEH1 and quantified the relative concentration of protein in the cytosol (light phase; C_L_) in *N. benthamiana*, as a proxy for protein saturation concentration (C_sat_) (Fig. 2a,b). Deletion of all individual domains resulted in significantly increased C_L_, implying that both IDRs and structured domains promote condensation, consistent with additional results from yeast (Extended Data Fig. 2a). Notably, deletion of IDR1 resulted in abnormally formed condensates, suggesting that IDR1 may function as a regulatory region (Fig. 2a).

To investigate the function of the AtEH1 IDRs, we conducted a sequence alignment of 128 AtEH1 homologs throughout 700 million years of plant evolution. Conservation analysis revealed that IDR1 and IDR2 are relatively divergent at the single amino acid level, while IDR3 is more conserved, likely due to the presence of short linear motifs which may facilitate protein interactions (Extended Data Fig. 2b). Analysis of homologous AtEH IDR1 sequences from plants, humans, and yeast showed they share similar sequence composition (Extended Data Fig. 2c), suggesting that the composition, but not the order of residues may be important for regulating condensate properties. To test this, we scrambled the IDR1 sequence of AtEH1, or replaced it with the corresponding IDR sequence from liverwort (MpEH1), yeast (ScPan1, ScEde1), or humans (HsITSN1). Scrambling, or replacement with MpEH1, HsITSN1, or ScEde1 IDRs resulted in cells with mostly regularly distributed condensates (Fig. 2c,d). Replacement with ScPan1 IDR induced irregularly distributed condensates (Fig. 2c,d), possibly due to a low proportion of basic residues (1.7%), compared to AtEH1 (4.9%) (Fig. 2e). Mutation experiments revealed that aromatic and basic residues, but not acidic or proline residues influence condensate properties (Fig. 2f,g). We further analysed the amino acid composition and sequence patterning features of AtEH1 IDR1 and homologous sequences using the NARDINI+ algorithm^36–38^ (Extended Data Fig. 2d). We found that plant EH proteins clustered separately from ScPan1 and ScEde1, which differed in the amount of charged residues, including arginine, supporting a role for charge in regulating AtEH1 condensate properties. Taken together, our results show that the composition, rather than the sequence order of residues regulates the properties of AtEH1-driven condensates, and that there is likely selective pressure to maintain IDR sequence composition within an optimal range.

### AtEH1 condensates nucleate via phospholipid interactions

**Extended Data Fig. 3 (Related to Figure 3):**
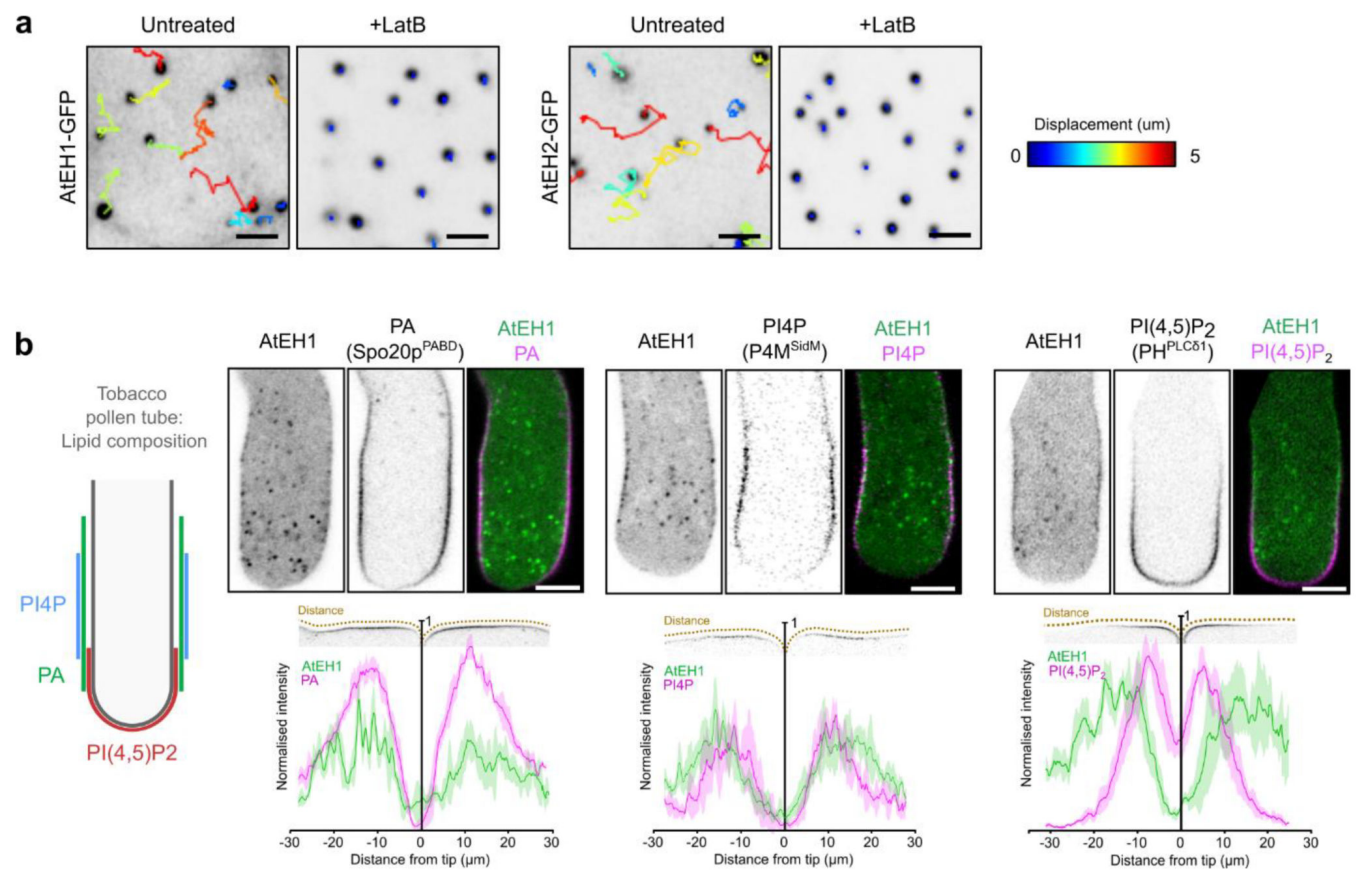
Condensate motility and phospholipid specificity experiments **a**, Representative time-lapse and tracking of AtEH1 and AtEH2 condensates in Latrunculin B treated (4 μM, 30 minutes) *N. benthamiana* epidermal cells. **b**, Schematic representation of the phospholipid localisation domains in Tobacco pollen tubes which maintain a distinctive lipid signature. Images show transient co-expression of pLAT52:AtEH1-YFP with lipid biosensors (pLAT52-biosensor-mCherry) in *N. tabacum* pollen tubes. The chart shows the quantification of the plasma membrane signal from AtEH1 and lipid biosensors from the tip of the pollen tube. Data represent mean ± SD from five time points from two individual pollen tubes. Scale bars = 5μm. For **a-b** the experiments were performed two times with similar results. Source data are provided as a Source Data file.

**Figure 3:**
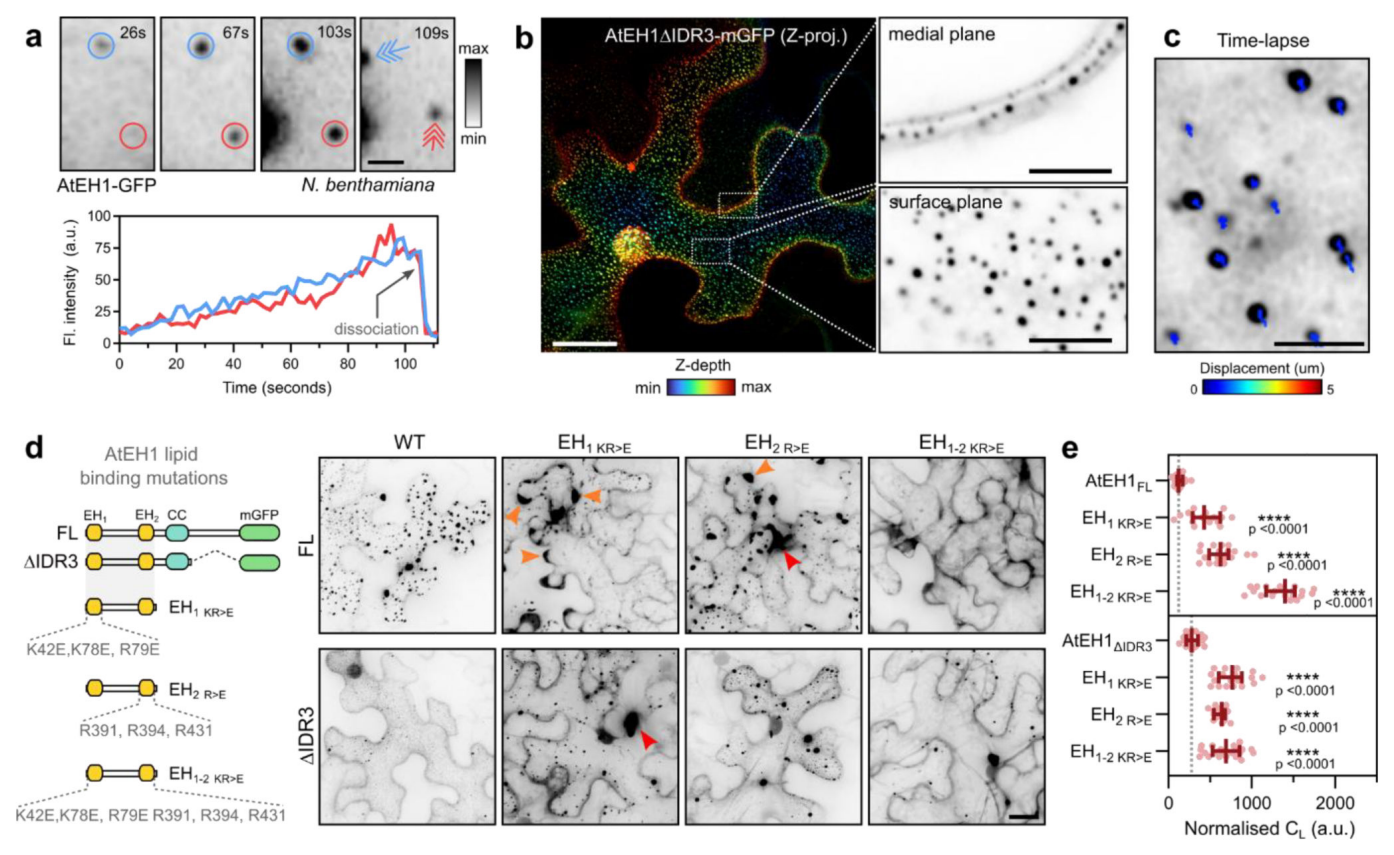
AtEH1 condensates nucleate on the plasma membrane via phospholipid binding domains **a**, Time-lapse imaging of UBQ10:AtEH1-GFP in *N. benthamiana* epidermal cells. Condensates appear as immobile plasma membrane associated puncta that gradually increase in intensity (blue and red circles), before dissociating from their original location (arrows). **b**, Depth colour-coded projections of UBQ10:AtEH1_ΔIDR3_-mGFP in *N. benthamiana* epidermal cells. Insets (single Z-plane sections) show that the condensates are restricted to the plasma membrane. **c**, Tracking of condensate displacement over 1 minute. **d**, Schematic of EH domain lipid binding mutants in AtEH1_FL_ and AtEH1_ΔIDR3_ constructs and representative confocal images of UBQ10:AtEH1-GFP reporter constructs expressed in *N. benthamiana* epidermal cells. Orange arrowheads indicate irregular condensates appearing at cell lobes, red arrowheads indicate condensates with abnormal morphology. Condensates were not observed in AtEH1_FL_ when both EH domains were mutated. **e**, Quantification of light phase (C_L_). Data is median ± 95% CI. Statistics indicates significance to the control (AtEH1_FL_ or AtEH1_ΔIDR3_), **** = p <0.0001, unpaired t-test with Welch’s correction; n = 18, 16, 18, 20, 17, 18, 16, 21 cells. Scale bars = 2 μm (**a**), 5 μm (**b**; inset), 20 μm (**c, d**). **See also** Extended Data Fig. 3; Video S1. Experiments were performed three times with similar results. Source data are provided as a Source Data file.

As AtEH1 is recruited to the plasma membrane during the early phase of endocytosis simultaneously with other TPC subunits^23^, we reasoned that the plasma membrane could act as a surface to lower the energy barrier for nucleation of condensation^39^. Supporting this, we observed individual nucleation events on the plasma membrane in *N. benthamiana*, with condensates gradually increasing in size before dissociating from the membrane (Fig. 3a and Video S1), likely through interaction with actin^11^ (Extended Data Fig. 3a). These nucleation events had intensity profiles that were inconsistent with those of endocytic foci^18,19^, and likely represent the transient accumulation of AtEH1 molecules on the membrane in this system. Furthermore, AtEH1 formed evenly distributed, immobile membrane-associated condensates when the IDR3 was truncated (Fig. 3b,c). Therefore, the plasma membrane acts as a surface to nucleate AtEH1 condensates.

Since AtEH proteins bind negatively charged anionic phospholipids via their EH domains^12^, we asked whether nucleation occurs via interaction with anionic phospholipids on the plasma membrane. To test this, we mutated the three positively charged arginine and lysine residues that mediate lipid binding in each individual EH domain^12^, and examined the effect on condensate formation (Fig. 3d). Mutation of individual EH domains significantly increased the cytosolic protein concentration, and caused irregularly shaped condensates to form (Fig. 3d,e). Combined mutation of both EH domains largely abolished condensate formation in the AtEH1_FL_ reporter, with only a few condensates remaining in the truncated AtEH1_ΔIDR3_ reporter, suggesting that other factors such as membrane-bound protein partners may also facilitate nucleation. We also found that AtEH1 was preferentially recruited to phosphatidic acid (PA) and phosphatidylinositol 4-phosphate (PI4P) enriched domains in *Nicotiana tabacum* pollen tubes, but not with domains containing phosphatidylinositol 4,5-bisphosphate (PI(4,5)P_2_) (Extended Data Fig. 3b). Similar localisation patterns of TPC subunits TPLATE and TML are reported in pollen tubes when expressed at endogenous levels^9,10^, suggesting that the PA/PI4P-dependent condensate nucleation of AtEH1, and the recruitment of TPC to membranes during endocytosis are analogous events.

### AtEH1 selectively sequesters endocytic proteins

**Extended Data Fig. 4 (Related to Figure 4):**
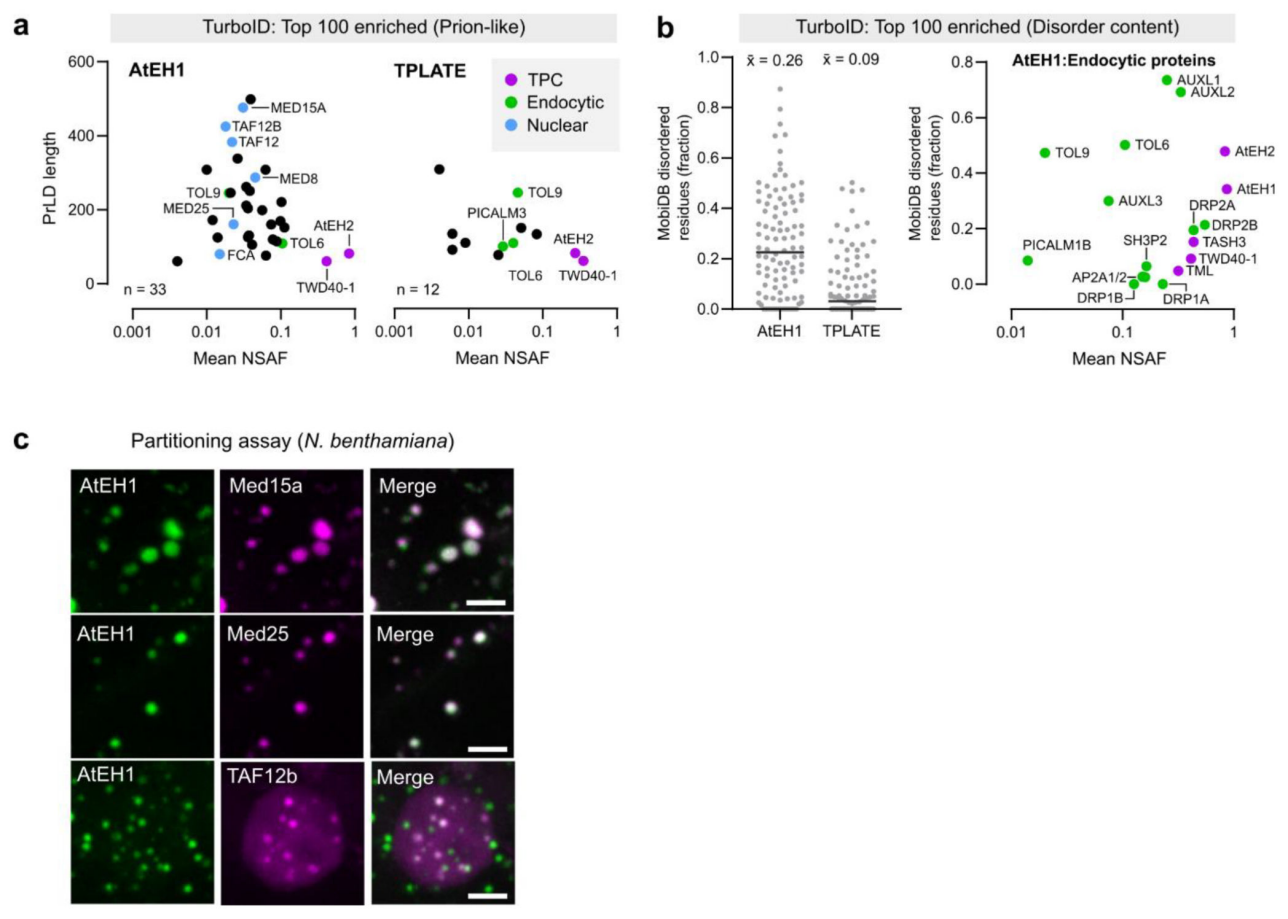
AtEH1 recruits proteins with prion-like domains **a**, Plot of Prion-like proteins identified in the AtEH1 and TPLATE TurboID datasets. **b**, (Left) Plot of top 100 enriched proteins based on disorder content (MobiDB), $\bar{x}$ (mean) disorder score is indicated. (Right) plot showing abundance and disorder content of selected endocytic proteins. **c**, Partitioning assay of selected nuclear proteins with prion-like domains identified in the AtEH1-TurboID dataset. UBQ10:AtEH1_FL_-GFP was co-expressed with mScarlet tagged client proteins in *N. benthamiana* epidermal cells. Partitioning was observed in the cytosol (Med15a, Med25) and in the nucleus (TAF12b). Scale bars = 5 μm (**c**). For **c** the experiment was performed two times with similar results. Source data are provided as a Source Data file.

**Figure 4:**
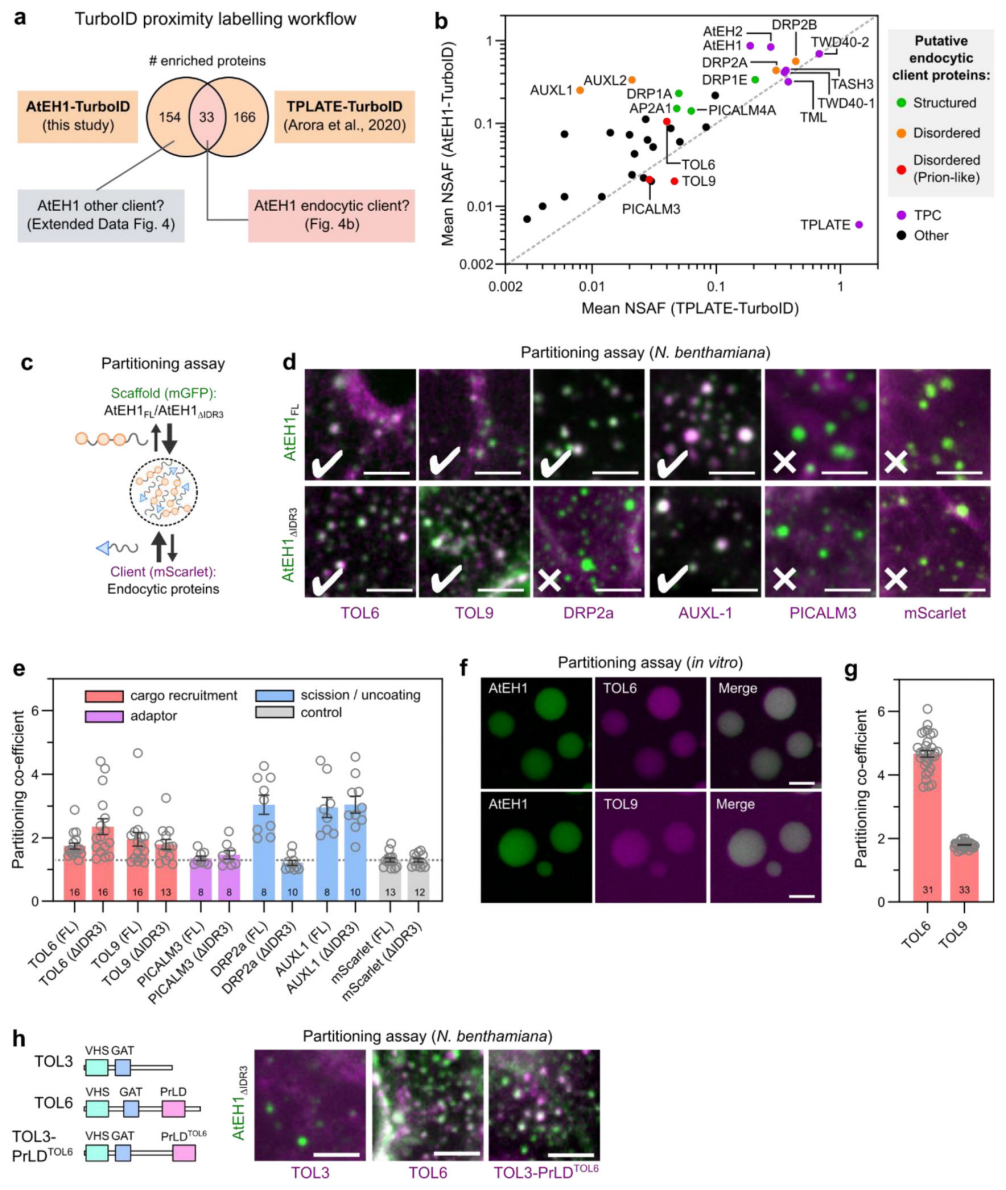
AtEH1 condensates selectively sequester endocytic machinery **a**, Interactomics strategy to identify endocytic client proteins of AtEH1. **b**, Enriched proteins in AtEH1 and TPLATE TurboID datasets were combined, and proteins common to both datasets are plotted. Auxilin-like1 and Auxilin-like2 were not enriched in the TPLATE-TurboID dataset, but included due to their high enrichment and abundance in the AtEH1-TurboID dataset. Proteins that function in endocytosis are coloured based on their disorder content. NSAF; normalised spectral abundance factor. **c**, Schematic of the partitioning assay. **d**, Representative images of scaffolds (AtEH1_FL_-GFP and AtEH1_ΔIDR3_-GFP) co-expressed with client proteins (Client-mScarlet) in *N. benthamiana* epidermal cells. **e**, Quantification of client partitioning. Values indicates partitioning co-efficient obtained for each cell. A partitioning coefficient of 1 indicates an absence of partitioning. Client proteins are coloured according to their function in endocytosis. Bars indicate mean ± SEM. The dashed line indicates the average background signal (mScarlet). **f,g** *in vitro* partitioning assay. Purified GFP-AtEH1 and TOL6-mCherry or TOL9-mCherry were combined. TOL6-mCherry and TOL9-mCherry did not phase separate individually. **f**, Quantification of client partitioning. Data indicate partitioning co-efficient from individual droplets. Bars indicate mean ± SEM. Numbers indicate the sample size (**e, g**). **h**, TOL chimera experiment. Representative images of TOL3, TOL6, or TOL3 fused with the Prion-like domain (PrLD) of TOL6 (mScarlet) co-expressed with AtEH1_ΔIDR3_-GFP in *N. benthamiana*. Scale bars = 5 μm. For **d-h** experiments were performed twice times with similar results. **See also** Extended Data Fig. 4. Source data are provided as a Source Data file.

Since AtEH subunits are multivalent proteins which drive condensate formation, we asked whether they function as scaffolds to passively recruit lower valency client proteins^40^. To identify potential client proteins, we performed TurboID proximity labelling proteomics in *A. thaliana* cell cultures using AtEH1 as bait. We compared the list of identified proteins with a previous TPLATE-TurboID dataset^41^ to differentiate between putative endocytic client proteins, and other proteins identified due to overexpression of AtEH1 (Fig. 4a). Endocytic proteins common to both datasets include the TOM-1LIKE (TOL) proteins TOL6 and TOL9, members of an ancestral ESCRT-0 complex which function as ubiquitin receptors at the plasma membrane^42–45^ (Fig. 4b). We also identified late phase endocytic proteins, including dynamins which mediate vesicle scission^46,47^, and AUXILIN-LIKE (AUXL) proteins, which function in vesicle uncoating^48^. We tested client recruitment using a partitioning assay in *N. benthamiana*, focusing on clients containing disordered regions which could potentially mediate weak, multivalent interactions with AtEH1 (Fig. 4b and Extended Data Fig. 4a,b). TOL6, TOL9 and AUXL1 readily partitioned into AtEH1 condensates, independently of the IDR3 of AtEH1. Conversely, DRP2a required IDR3 for partitioning (Fig. 4c-e), indicating that IDR3 can facilitate protein interactions. PICALM3 did not partition, and may require additional factors such as membrane curvature^49^. Additionally, we demonstrated a direct interaction between AtEH1 and TOL6/TOL9 by partitioning assays *in vitro* (Fig. 4f,g).

Intriguingly, we found that the AtEH1-TurboID dataset was enriched with disordered and prion-like proteins (Extended Data Fig. 4a,b), suggesting that AtEH1 may interact with PrLDs with specific features. Supporting this, we observed partitioning of nuclear prion-like proteins identified as highly enriched in the AtEH1-TurboID dataset (Extended Data Fig. 4c). Furthermore, transplanting the PrLD of TOL6 onto TOL3 (which lacks a PrLD) was sufficient to promote partitioning with AtEH1 (Fig. 4h), demonstrating that AtEH1 can recruit proteins via weak, multivalent interactions. Collectively, these data demonstrate that AtEH1 is an endocytic scaffold which can selectively recruit cytosolic accessory proteins throughout endocytosis.

### AtEH1 condensates enable clathrin recruitment and assembly

**Extended Data Fig. 5 (Related to Figure 5):**
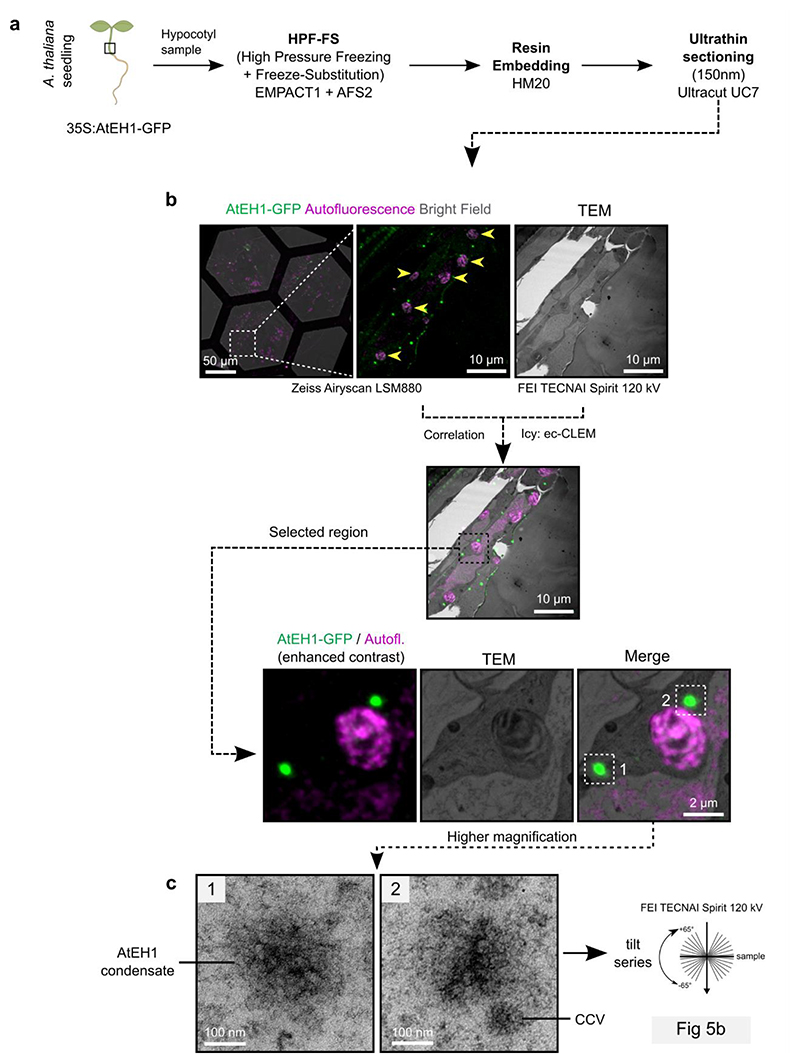
CLEM-ET workflow **a**, Overview of the sample preparation procedure. **b**, Expanded workflow of the Correlative Light and Electron Microscopy (CLEM) imaging of hypocotyl sections. Yellow arrowheads indicate chloroplasts used as natural landmarks for correlation. **c**, Zoomed images of regions selected for electron tomography (ET) reconstruction. The condensates have similar electron density to CCVs.

**Figure 5:**
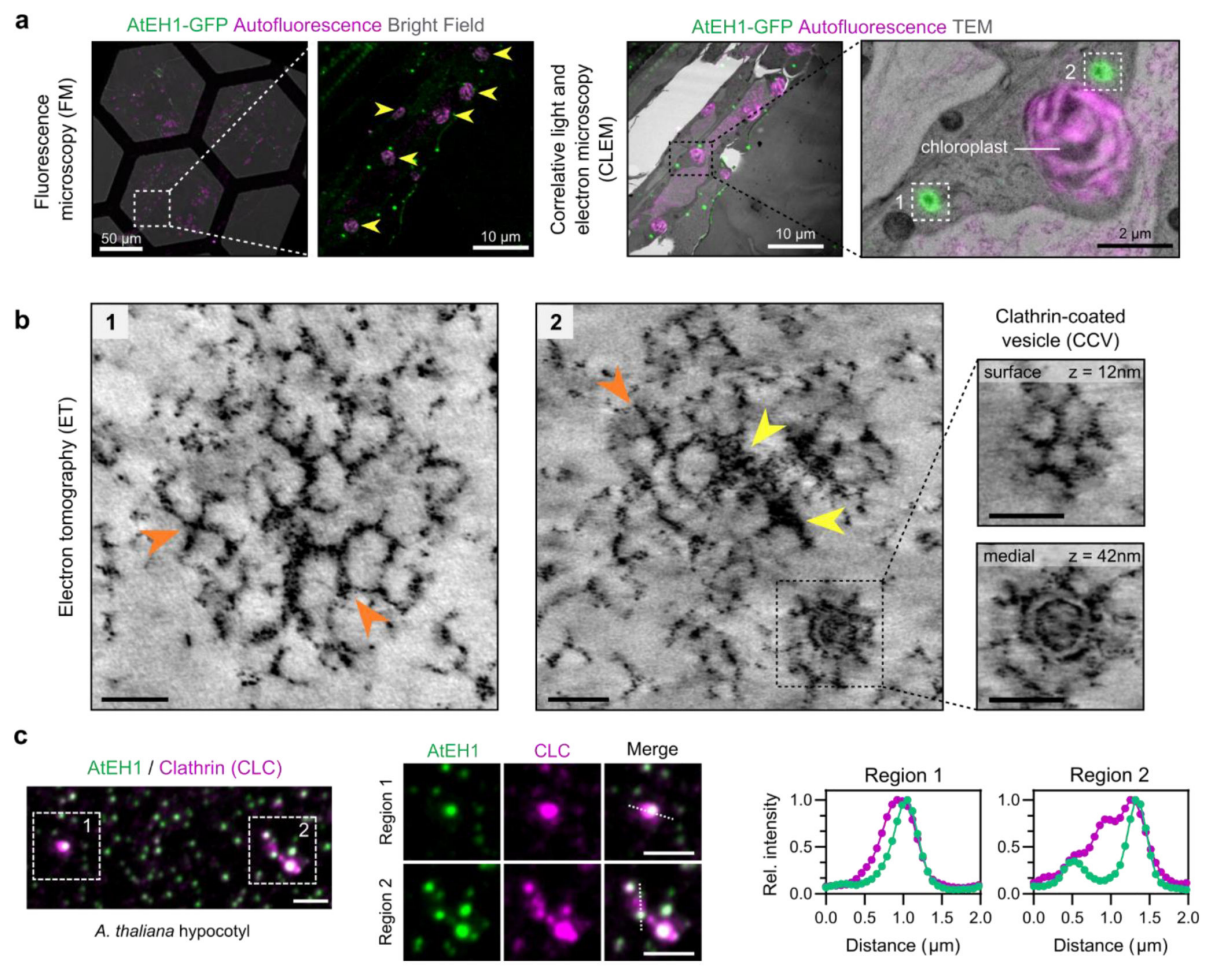
Condensation of AtEH1 facilitates clathrin recruitment and assembly **a**, Fluorescence and electron microscopy images of hypocotyl resin sections from *A. thaliana* seedlings over-expressing AtEH1 (35S:AtEH1-GFP). Yellow arrowheads indicate chloroplasts used as natural landmarks for correlation. **b**, Electron tomography (ET) reconstructions of the selected regions from **a**. Clathrin-like lattices and triskelia (orange arrowheads), and electron dense accumulations (yellow arrowheads) are formed within AtEH1 condensates. Inset shows a clathrin-coated vesicle (CCV) nearby the condensate. **c**, Representative airyscan images of clathrin light chain 2 (CLC-mKo) and AtEH1 (35S:AtEH1-GFP) in *A. thaliana* hypocotyl cells. Plot profiles show normalised fluorescence intensities from the indicated dashed lines. Clathrin is observed within and surrounding the condensates. Scale bars = as indicated (**a**), 50 nm (**b**), 2 μm (**c**). **See also** Extended Data Fig. 5; Video S2. Source data are provided as a Source Data file.

Endocytosis involves the assembly of endocytic accessory proteins and the coat protein clathrin to ordered basket-like assemblies on the plasma membrane. Because condensation of AtEH1 is sufficient for the recruitment of endocytic accessory proteins, we asked if we could observe structural arrangements within AtEH1 condensates. To achieve this, we combined correlative light and electron microscopy (CLEM) with electron tomography (ET) to examine the ultrastructure of AtEH1-GFP-driven condensates in a stable *A. thaliana* overexpression line (Fig. 5a, Extended Data Fig. 5a,b). We identified large cytosolic condensates which had a similar electron density as neighbouring clathrin-coated vesicles (CCVs) (Extended Data Fig. 5c). Tomography reconstruction revealed the presence of ordered polygonal cage-like assemblies which resemble clathrin triskelia and partial clathrin cages (Fig. 5b, Video S2). Furthermore, clathrin readily partitioned into these condensates (Fig. 5c), supporting our view that the ultrastructure assemblies we observe are composed of clathrin. These findings demonstrate that condensation of AtEH1 is sufficient to recruit clathrin and facilitate its re-arrangement into ordered assemblies.

### TPC drives condensation and clathrin assembly on membranes

**Extended Data Fig. 6 (Related to Figure 6):**
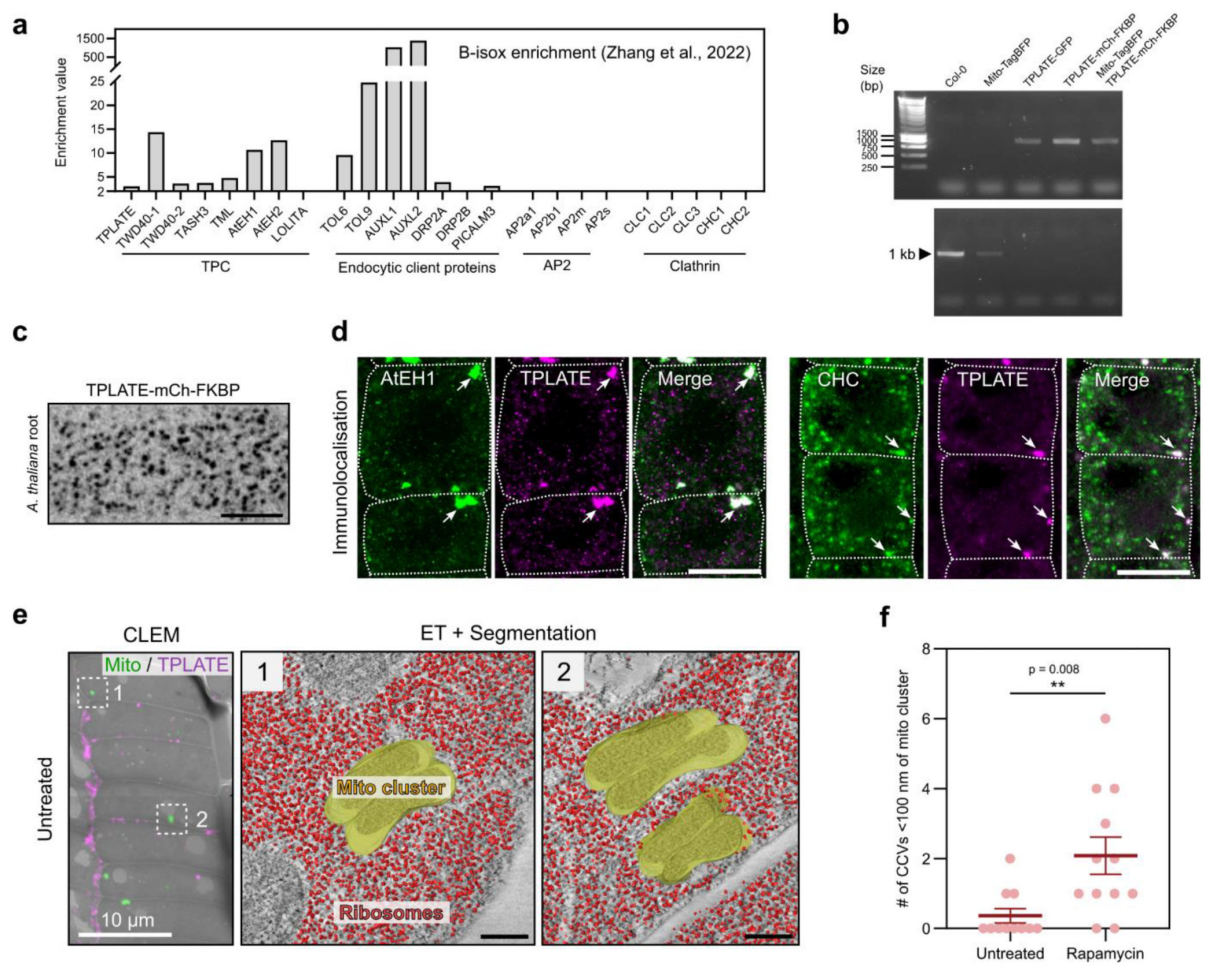
B-isox enrichment and TPLATE re-localisation additional data **a**, Enrichment of TPC subunits and endocytic proteins after b-isox treatment. Data shown is from Zhang et al., 2022. Proteins with enrichment values ≥ 2 are considered enriched, and < 2 not enriched. Enrichment values below 2 were not represented in Zhang et al., 2022. **b,c**, Validation of TPLATE-mCh-FKBP *tplate A. thaliana* lines. Genotyping PCR indicate TPLATE-mCh-FKBP complements the *tplate* mutant (**b**). Primers; Top row: *tplate T-DNA*, bottom row: WT TPLATE. TPLATE-GFP *tplate*(-/-) (positive control). Endocytic foci from *A. thaliana* root cells expressing TPLATE-mCh-FKBP (**c**). **d**, Whole mount immunolocalisation of AtEH1 (Anti-AtEH1), CHC (Anti-CHC), and TPLATE-FKBP-mCherry (Anti-RFP) in rapamycin treated *A. thaliana* root cells. Arrows indicate mitochondria clusters. **e**, CLEM localisation and ET of mitochondria clusters from untreated *A. thaliana* root cells. No ribosome exclusion zone between mitochondria clusters was observed. **f**, Quantification of CCV recruitment to mitochondria clusters from tomograms from control (n=11) and rapamycin treated (n=12) root cells obtained from n = 2, and 2 biologically independent samples. Bars indicate mean ± SEM. ** p<0.01; unpaired two-tailed t-test. For **b-d** the experiments were performed two times with similar results. Scale bars = 5 μm (**c**), 10 μm (**d**), 200 nm (**e**), or as indicated. Source data are provided as a Source Data file.

**Figure 6:**
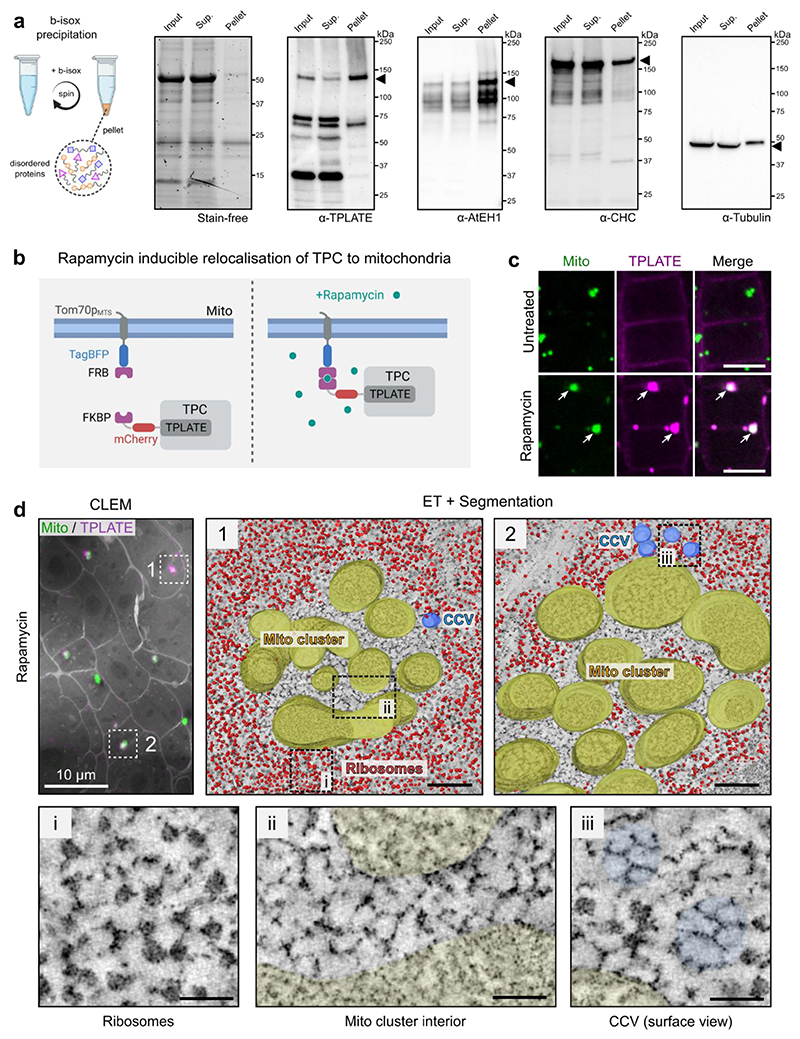
Chemical induced re-localisation of TPC to mitochondria is sufficient for condensate formation and clathrin assembly **a**, Biotinylated isoxazole (B-isox) enrichment assay was used to confirm the enrichment of TPC subunits in the pellet fraction enriched for disordered proteins. The input, supernatant, and pellet fractions were used for western blots with the indicated antibodies. The stain free gel shows selective enrichment of proteins in the pellet fraction. Tubulin and clathrin (CHC) are structured proteins which are not enriched in the pellet fraction. **b**, Schematic of the chemically induced TPLATE mitochondria re-localisation. **c**, Images of *A. thaliana* root cells expressing Tom70p_MTS_-TagBFP2-FRB (Mito) and TPLATE-mCherry-FKBP (TPLATE) from untreated, and rapamycin-treated seedlings. TPLATE is re-localised to mitochondria clusters after rapamycin treatment (arrows). **d**, CLEM localisation and segmented ET reconstruction of mitochondria clusters from a rapamycin treated *A. thaliana* root cells (see also Extended Data Fig. 6). Insets show that the interior of the mitochondria cluster is devoid of ribosomes and contains clathrin lattice-like assemblies. Scale bars = 10 μm (**c**), 200 nm (**d**), 50 nm (**d-inset**). **See also** Extended Data Fig. 6; Video S3 and S4. For **a** and **c** experiments were performed two times with similar results. Source data are provided as a Source Data file.

Our previous work suggests that TPC exists as a stable complex in the cytosol, which is recruited collectively to the plasma membrane during the initiation of endocytosis^13,23^. The small molecule biotinylated isoxazole (b-isox) has been shown to precipitate proteins containing disordered regions, including those found in condensates such as RNA granules^50^. Both structured (e.g. TPLATE, TWD40-1) and disordered (AtEH1, AtEH2) TPC subunits were selectively precipitated in a large scale b-isox experiment in *A. thaliana* (Extended Data Fig. 6a)^51^. We independently confirmed that AtEH1 and TPLATE are enriched in b-isox precipitates, while clathrin and tubulin were not (Fig. 6a). These data imply that structured TPC subunits precipitate because they exist in a highly stable complex together with the disordered AtEH subunits, and consequently the whole TPC participates in condensate formation.

We therefore reasoned that membrane-driven concentration of the octameric TPC may trigger condensation as a complex. To test this, we used an inducible FRB-FKBP rapamycin-based interaction system to concentrate TPC at endogenous stoichiometric ratio on mitochondrial membranes *in vivo* (Fig. 6b and Extended Data 6b,c)^52,53^. After rapamycin addition, TPLATE rapidly re-localised to mitochondria clusters (Fig. 6c and Video S3). These clusters contained AtEH1 and clathrin, confirming that other TPC subunits and interacting proteins were recruited (Extended Data Fig. 6d). CLEM-ET revealed a distinct ribosome exclusion zone in the interior of TPC positive mitochondria clusters, which were not observed in the untreated control (Fig. 6d and Extended Data Fig. 6e,f). This zone contained ordered, clathrin lattice-like assemblies which spanned most of the area (Fig. 6d and Video S4). We also observed significantly more clathrin-coated vesicles nearby TPC-positive mitochondria clusters compared to the non-induced control cells (Fig. 6d, Extended Data Fig. 6f), in agreement with TPC condensates having an affinity for clathrin (Extended Data Fig. 6d). Together, these data show that concentration of TPC on a membrane *in vivo* is sufficient to generate condensates which promote clathrin recruitment, possibly through collective interactions between clathrin, TPC, and other endocytic machinery.

### Condensate material properties affect endocytic progression

**Figure 7:**
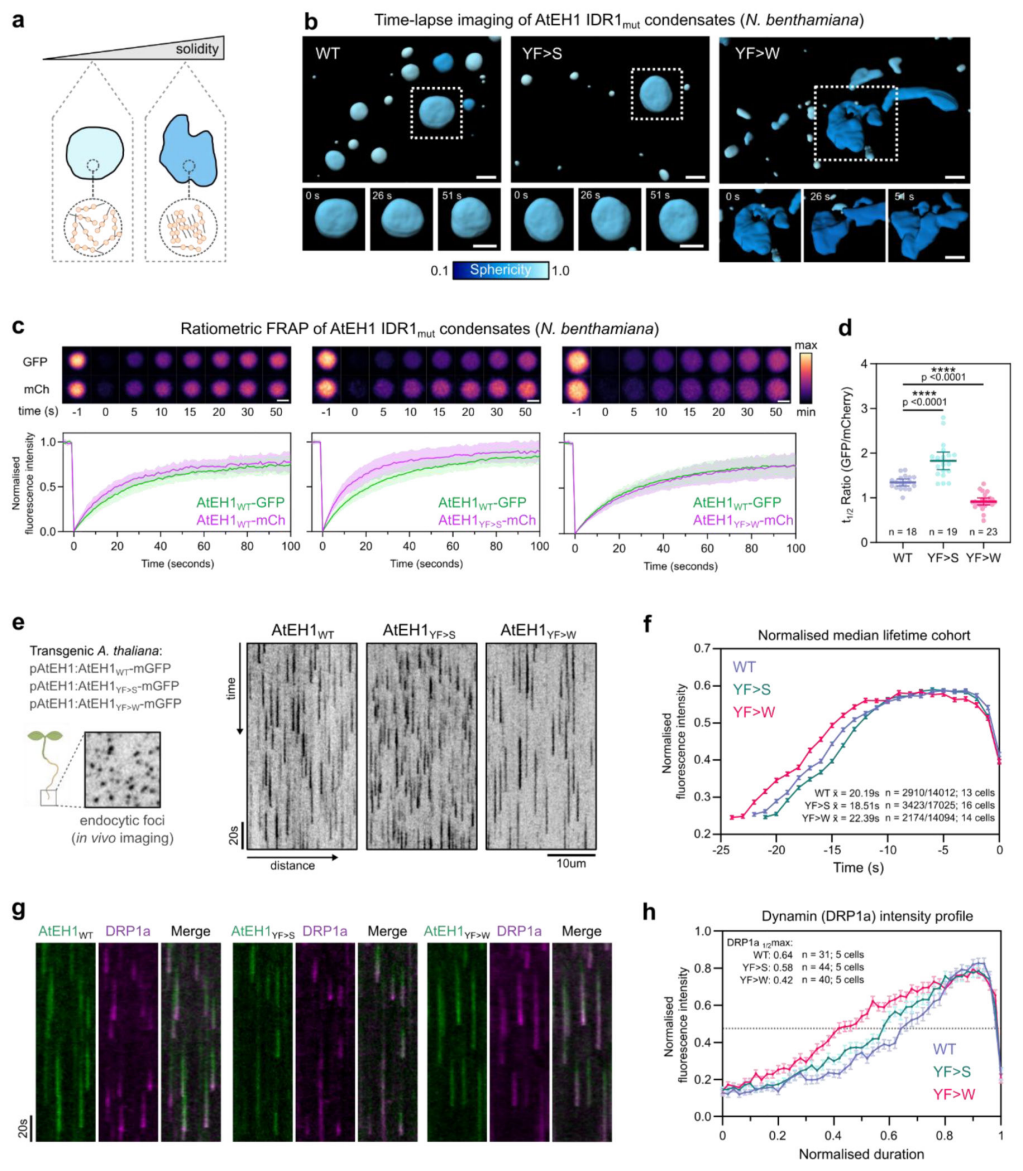
The material properties of TPC-driven condensates are important for the progression of endocytosis **a**, Schematic representation of condensate material properties. **b**, 3D surface rendering of condensates from *N. benthamiana* epidermal cells expressing UBQ10:AtEH1_WT/mut_-GFP constructs. Condensates are coloured by sphericity, with a value of 1 indicating a perfect sphere. **c**, Representative ratiometric FRAP images of condensates from *N. benthamiana* epidermal cells. Plot shows FRAP recovery curves from a binned subset of the data (n= 8); plot indicates mean ± SD. **d**, Quantification of t_1/2_ ratio from all FRAP curves; n indicates the number of condensates analysed from n = 11, 12, and 12 biologically independent cells. Bars indicate mean ± 95% CI. Statistics indicate difference from WT; **** p<0.0001, unpaired t-test with Welch’s correction. **e**, Kymograph analysis of endocytic events from root cells of *A. thaliana eh1-1* homozygous mutant lines rescued by AtEH1:AtEH1_WT/MUT_-GFP. **f**, Mean fluorescence intensity calculated from the median lifetime of endocytic events; error bars indicate SEM. The mean lifetime, the number of endocytic events in the median lifetime cohort, and the total number of measured events is indicated. **g**) Kymograph analysis of endocytic events from root cells of *A. thaliana* AtEH1:AtEH1-GFP/*eh1-1* lines in a DRP1a-mRFP/*drp1a* background. **h**) Mean DRP1a fluorescence intensity profiles obtained from kymograph analysis (see also Extended Data Fig. 7g). Error bars indicate SEM. The duration to reach half maximum DRP1a intensity values (dashed line) is indicated. Data in **c-d** and **e-f** represents pooled data from two and three independent repeats respectively. The experiment in **g-h** was performed three times with similar results. Scale bars = 5 μm (b), 2 μm (c), 10 μm (e). **See also** Extended Data Fig. 7; Video S5. Source data are provided as a Source Data file.

We next asked whether condensation of TPC is essential for functional endocytosis *in vivo*. Aromatic residues in PrLDs have been shown to influence protein phase separation^33,54^, including affecting the material properties of condensates in plants^55^. We therefore reasoned that we could alter TPC-driven condensates by modifying aromatic residues in the prion-like IDR1 of AtEH1. We used the modified AtEH1 reporters where we substituted the weaker interacting residues (phenylalanine and tyrosine) with stronger (tryptophan) residues (12YF>W), or mutated them to serine (12YF>S) to abolish aromatic-based interactions (Fig. 2f). We inferred the material properties of these condensates by high speed 4D imaging in *N. benthamiana*. AtEH1_YF>W_ condensates were highly dynamic and irregularly shaped, indicating altered viscoelasticity compared to AtEH1_YF>S_ and AtEH1_WT_ condensates, which were more spherical (Fig. 7a,b, and Video S5). Consistent with these data, quantification of molecular exchange using a ratiometric FRAP assay revealed that increasing aromatic interaction strength (AtEH1_YF>W_) reduces protein exchange (Fig. 7c,d). These results demonstrate that the IDR1 interaction strength of AtEH1 influences the resulting dynamics and material properties of these condensates.

**Extended Data Fig. 7 (Related to Figure 7):**
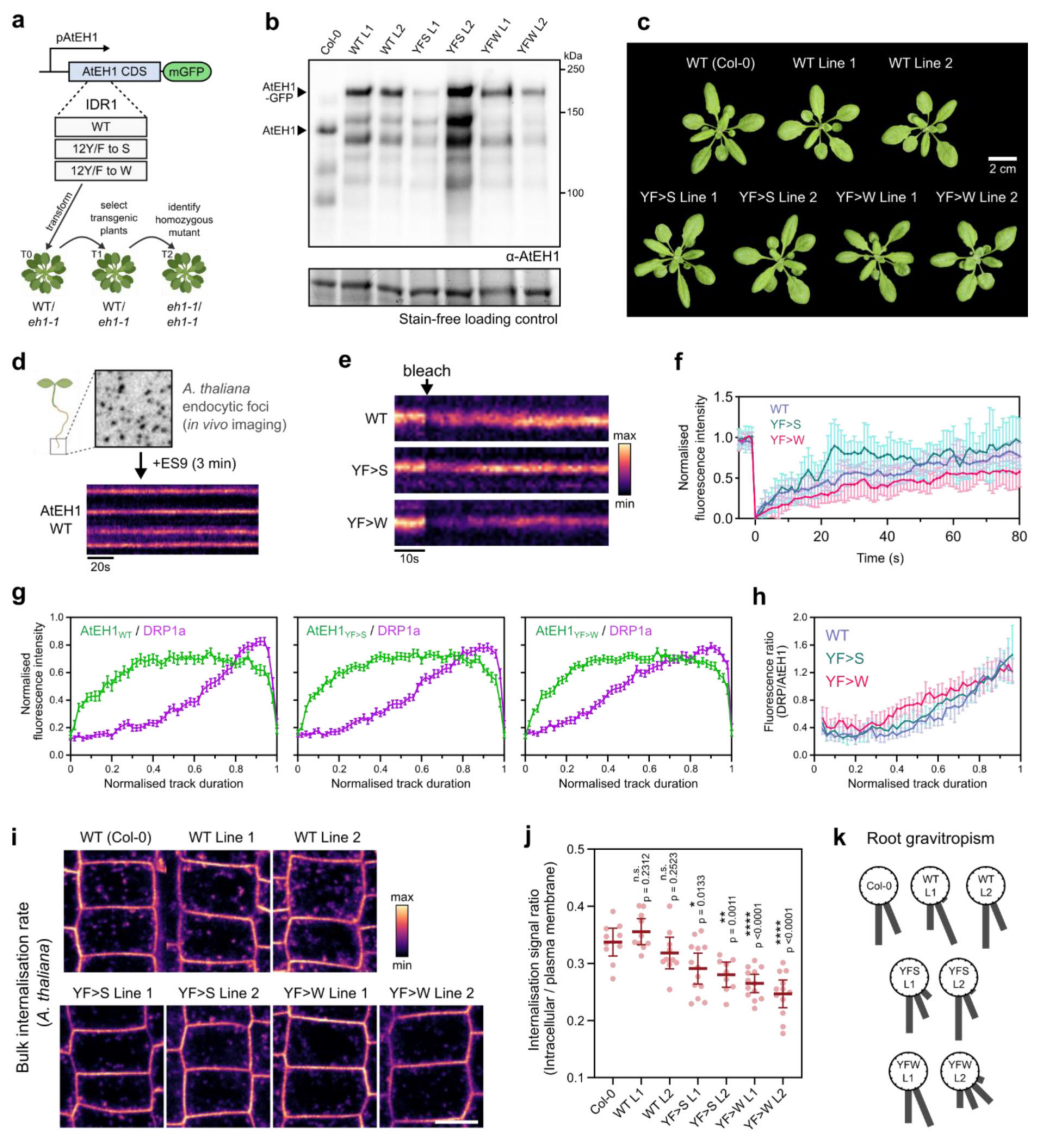
pAtEH1:AtEH1-GFP reporter validation and functionality **a**, Schematic of the generation of pAtEH1:AtEH1-GFP rescue lines. **b**, Western blot of *A. thaliana* protein extracts using an antibody directed against AtEH1, indicating absence of endogenous AtEH1 in the recued lines. Lower molecular weight bands are degradation products. Strain free gel is shown as a loading control. **c**, Representative plant rosette image of WT Col-0 and AtEH1 rescued lines. **d-f**, Inhibition of endocytosis in pAtEH1:AtEH1-GFP seedlings using the chemical inhibitor ES9 (3 min, 10 μM). Kymograph shows immobile endocytic foci with stable fluorescence intensity over time (**d**). Kymograph (**e**) and fluorescence intensity plot (**f**) from FRAP experiments of individual endocytic foci from pAtEH1:AtEH1_WT_-GFP, pAtEH1:AtEH1_YF>S_-GFP, and pAtEH1:AtEH1_YF>W_-GFP seedlings. Plotted values indicate mean ± SD. Half recovery times (t_1/2_) are 22.08s, 11.95s, 28.81s, respectively. **g**, Full intensity profiles from kymograph analysis of endocytic events from root cells of *A. thaliana* AtEH1:AtEH1-GFP/*eh1-1* lines in a DRP1a-mRFP/*drp1a* background (see also Fig. 7g,h). Plotted values indicate mean ± SEM. **h**, Fluorescence intensity ratio of DRP1a compared to AtEH1 from data in (**g**). Plotted values indicate median ± 95% CI. (**i,j**) Endocytic flux experiment. Seedlings were treated with the lipophilic dye FM4-64 (2μM, 10min) and root cells were imaged (**i**). **j**, Quantification of FM4-64 internalisation. Bars indicate mean ± 95% CI. Statistics indicate significance to WT; n.s. not significant, * p<0.05, ** p<0.01, **** p<0.0001, unpaired t-test. **k**, Gravitropism assay. Roots grown on ½ MS agar plates were gravistimulated by turning them 90°. The gravitropism plot indicates the angle of the root tip in 22.5° bins. n= 20 roots for each genotype. Scale bar = 10 μm (**d**). Data in **i-j** represents pooled data from three independent repeats. The experiment in **b, f** and **k** was performed two, two and three times, respectively with similar results. Sample sizes (**f, j, k**) are indicated in Source Data. Source data are provided as a Source Data file.

To investigate the effect on endocytosis in *A. thaliana*, we generated native promoter (pAtEH1:AtEH1-mGFP) wild type (WT), and aromatic mutant (YF>S, YF>W) reporters. These lines contained close to endogenous levels of AtEH1 protein, and were functional as they rescued the *A. thaliana* male sterile *eh1-1* mutant (Extended Data Fig. 7a-c). Molecular exchange within chemically stalled endocytic foci *in A. thaliana* root cells correlated with the reported differences in fluorescence recovery in *N. benthamiana* (Extended Data Fig. 7d-f), confirming that modification of IDR interaction strength influences condensate material properties at near-to endogenous expression levels. Live cell imaging and automated analysis of endocytosis lifetime revealed an increase in endocytic lifetime with increasing interaction strength (AtEH1_YF>W_), and a shorter lifetime when interaction strength was decreased (AtEH1_YF>S_) (Fig. 7e,f), likely indicating impaired endocytosis in these lines.

To determine whether the timing or recruitment of endocytic accessory proteins were altered, we combined these reporters with the dynamin-related protein DRP1a, a late-stage scission protein, which interacts with TPC^9^. DRP1a showed a characteristic late-stage recruitment profile in AtEH1_WT_, contrasting with a relatively linear recruitment profile in the AtEH1_YF>W_ line, while AtEH1_YF>S_ showed an intermediate profile (Fig. 7g,h and Extended Data Fig. 7g,h). These data indicate that the recruitment and re-arrangement of endocytic machinery are impaired by modifying TPC-driven condensate material properties, and this impairs endocytic progression and efficiency. Consistently, bulk endocytic internalisation was strongly impaired in AtEH1_YF>W_ seedlings (Extended Data Fig. 7i,j), indicating reduced endocytosis. Furthermore, the disruption to endocytosis also had a physiological effect, as we observed delayed root gravitropism responses in AtEH1_YF>W_ seedlings (Extended Data Fig. 7k), a process which requires TPC-dependent endocytic activity^9^. Collectively, these findings show that the IDR composition of endocytic machinery impacts the emergent physical properties of TPC-driven condensates, which are essential for functional endocytosis.

## Discussion

Here, we discovered that biomolecular condensation of the plant TPC promotes the recruitment and assembly of clathrin and accessory proteins, from the initiation of endocytosis to vesicle scission. The biophysical properties of these condensates are encoded in the sequence chemistry of disordered regions in the AtEH TPC subunits, and are essential for efficient endocytosis *in vivo*. This study demonstrates how weak, multivalent interactions collectively drive condensation and facilitate the dynamic re-arrangement of a meshwork of proteins during endocytosis^56^.

Our work highlights a connection between membrane-driven condensate nucleation and the initiation of endocytosis *in vivo*. Our *in vivo* CLEM data shows that concentration of TPC on a membrane is sufficient to trigger condensation, likely by limiting molecular diffusion to a two-dimensional plane which reduces the nucleation energy barrier^39,57^. Condensation facilitates the downstream recruitment of adaptors and clathrin to initiate the assembly of clathrin-coated pits. Consequently, the nucleation of condensation must be highly regulated. Our data indicate that specificity is primarily generated through interaction of AtEH1 with PA and PI4P^12^, two key phospholipids implicated in endocytosis in plants^58,59^. In contrast, condensation of Eps15/FCHo in animals is dependent on PI(4,5)P_2_^25^. Interaction of TPC with PI4P via the muniscin-like subunit TML may further enhance recruitment specificity^13^, or could promote complex stability at the membrane, similar to the role of FCHo^24,30,31^. Further specificity may be generated through recognition of ubiquitinated cargo by TOM1-LIKE (TOL) proteins^44,45,60^, or via the TPC subunit TASH3^16^. This coupling of recruitment and condensation of TPC to the recognition of lipids and ubiquitinated cargo would ensure the highly regulated assembly of clathrin-coated vesicles on the plasma membrane. Similar, lipid— or receptor—mediated nucleation mechanisms may promote the selective assembly of other membrane-driven condensates, including during autophagosome formation^61^.

Our CLEM-ET experiments indicate that condensation of TPC promotes the recruitment and assembly of clathrin during endocytosis. During the dynamic growth phase of clathrin coated pits, clathrin is rapidly deposited at the edge of the growing lattice^3,4^. AtEH homologs Eps15, FCHO, Intersectin and Ede1p are spatially restricted to the lattice edge^28,49,62^, similar to the model proposed for TPC^18^. Recruitment of clathrin by TPC subunits^9,15^ to the condensate may promote the sustained assembly of clathrin into the growing lattice, and could explain why TPC arrives before clathrin on the membrane^9,19^. Notably, condensates facilitate the exchange of protein with the cytosolic pool^32^, and condensation could therefore be a mechanism which reconciles the rapid exchange of clathrin observed throughout endocytosis^63^. Furthermore, TPC likely promotes the assembly of dynamin and auxilin at the neck of invaginated clathrin coated pits during late endocytosis, consistent with a function for TPC throughout endocytosis^9,23^. Given that Ede1p and Eps15/FCHo are absent from the late phase of endocytosis, late-stage condensation in animals and yeast may be driven by discrete proteins including the middle-late arriving proteins Sla1p^64^, and human endophilin^65^, rather than via a stable complex as we show in plants.

Finally, we identified IDRs as regulators of endocytosis. The physical properties of condensates are controlled by multivalent, co-operative interactions between molecules which form a physically cross-linked network^66^. Our results demonstrate that interactions via the IDR1 of AtEH1 influence the physical properties of condensates, thereby affecting the recruitment of accessory proteins and endocytosis efficiency *in vivo*. Given that the IDR composition of plant AtEH proteins shows similarities, but also distinct features compared to yeast and animal homologs, there is likely an evolutionary optimal physiochemical environment derived from collective IDR properties which provide a balance between ordered (solid-like) and flexible (liquid-like) network assemblies during endocytosis. Notably, artificially strengthening Eps15 dimerisation impairs endocytosis in human cells^25^, highlighting that multivalency of endocytic scaffold proteins driven by both dimerisation and IDR-mediated interactions are essential aspects of endocytosis. Distinct IDR sequence grammar can determine both selectivity of partner recruitment and condensate formation^38^. How recruitment of endocytic accessory proteins, condensate formation as well as membrane binding is encoded into the IDR of AtEH1 and other endocytic protein IDRs remains unclear.

In summary, we have identified biomolecular condensation as a fundamental organisation principle promoting the assembly of clathrin and endocytic accessory proteins throughout endocytosis. Our findings have implications for understanding how membrane-driven condensates form and act as organisation hubs to generate selective and dynamic interaction networks.

## Methods

### Plant material and growth conditions

*Arabidopsis thaliana* accession Columbia-0 (Col-0) plants were used for all experiments. Seeds were surface sterilised by chlorine gas and grown on ½ strength Murashige and Skoog (½ MS) medium containing 0.6% (w/v) agar, pH 5.8, without sucrose. Seedlings were stratified for 48 h at 4°C in the dark, and transferred to continuous light conditions (68 μE m−2 s−1 photosynthetically active radiation) at 21 °C in a growth chamber. Imaging was performed on 4–5-day old seedlings unless otherwise indicated. β-Estradiol induction of the pRPS5A:XVE lines was performed by transferring 3–day-old seedlings to ½ MS medium containing 1 µM β-estradiol. Plant materials are described in the Reporting Summary.

### Chemical treatments

Chemical stock solutions of β-Estradiol (20 mM in DMSO), ES9^67^ (10 mM in DMSO), Latrunculin B (4 mM in DMSO), and Rapamycin (10mM in DMSO) were used at the concentrations indicated. Seedlings were incubated in 6-well plates in ½MS with the indicated chemicals. For the rapamycin experiments, seedlings were shaken at 100 rpm.

### Molecular cloning

All constructs were cloned and assembled using the Green Gate cloning system unless otherwise indicated and detailed in Table S1. Entry vectors were assembled by Gibson assembly using NEBuilder® HiFi DNA Assembly.

### Generation of *A. thaliana* reporter lines

To generate the pAtEH1:AtEH1-GFP reporters, a 2356 bp promoter and 5’UTR region was fused to the CDS of AtEH1 and mGFP using Golden Gate cloning. Transformation was performed by floral dip into plants heterozygous for the *eh1-1* T-DNA (SALK 083997). Positive transformants were selected by spraying with Basta solution on seedlings grown in soil. Heterozygous *eh1-1* plants were selected, and in the following generation seedlings homozygous for *eh1-1* were identified by genotyping and confirmed via Western blot. To generate the pH3.3-MITOTagBFP2-FRB pLAT52-TPLATE-mCherryFKBP *tplate* line, plants heterozygous for the *tplate* T-DNA (SALK 0030086) were transformed with pLAT52-TPLATE-mCherryFKBP by floral dip. T1 Plants were selected with 25 mg/L hygromycin for presence of pLAT52::TPLATE-mCherry-FKBP, and genotyped to select *tplate(+/-)*. T2 plants were screened to identify *tplate* (-/-) plants. The following T3 seedlings were transformed with pH3.3::MITO-TagBFP2-FRB*. T1 plants were selected with 10 mg/L Basta, and plants homozygous for pH3.3-MITOTagBFP2-FRB, pLAT52-TPLATE-mCherryFKBP, and *tplate* were identified in the following generations. Lines containing pAtEH1:AtEH1-GFP (*eh1-1)* and DRP1a-mRFP (*drp1a*) were generated by crossing, and genotyped as homozygous for *eh1-1* and *drp1a* in the following generations.

### Gravitropism assay

5 day old *A. thaliana* seedlings were grown on ½ MS media. Seedlings of similar sizes were first transferred to a fresh ½ MS plate to reduce variability due to potential germination rate differences, and then gravistimulated by turning the plate 90° for 8 hours. Images were acquired every 15 minutes using a Canon EOS camera. The root tip angle was calculated in Fiji using the angle tool, and the values were placed into 22.5° bins.

### Transient expression in Tobacco epidermis, imaging, and analysis

*N. benthamiana* plants were grown in a greenhouse under long-day conditions (6–22 h light, 100 PAR, 21 °C) in soil (Saniflor osmocote pro NPK: 16-11-10 + magnesium and trace elements). Transient expression was performed by leaf infiltration using GV3101 Agrobacterium strains containing plasmids of interest. An agrobacterium strain containing the p19 silencing inhibitor was co-infiltrated for all experiments. Transiently transformed *N. benthamiana* were imaged three to four days after infiltration. For the analysis of AtEH1 truncations or mutations the relative saturation concentration was obtained by quantifying the mean cytosolic signal after subtraction of background signal. The partitioning assay quantification was performed using the MitoTally script using regions of interests determined from AtEH1 positive foci^52^.

### Pollen tube transformation, imaging, and analysis

Pollen tube experiments were performed as previously described^68^. Transformed tobacco (*Nicotiana tabacum*) pollen grains were observed 6 hours after transformation with a Zeiss LSM900 microscope in Airyscan 2 4Y multiplex mode using a 40x W 1.2 NA Plan Apo objective. Time-lapse images were acquired for 30-90s.

Analysis of plasma membrane intensities was performed in Fiji. A region of interest (ROI) was selected with the “Segmented line” tool over the plasma membrane region of the middle section of the pollen tube for each time point. For each time point, the AtEH1 (green) and biosensor (red) channel intensities were plotted. An offset distance value was manually calculated for each time point to align each time point to the centre of the pollen tube tip. The combined average of each channel was calculated from a total of 10 images.

### Yeast expression

Fission yeast strain LD328 was used in this study. Cells were transformed by the standard LiAc/SS carrier DNA/PEG method. Transformants were plated on EMM+HT medium (EMM medium supplemented with 45mg/L histidine and 15 µM thiamine). After 3 days, colonies were transferred to EMM+H (EMM medium supplemented with 45mg/L histidine) plates and observed with a Zeiss LSM880 confocal microscope using a 100x Oil 1.4 NA objective.

### *In vitro* protein expression and purification

All proteins were expressed in Escherichia coli BL21 (DE3) cells. Briefly, protein expression was induced by 0.5 mM isopropyl β-D-1-thiogalactopyranoside (IPTG) for 18 h at 18°C. Cells were collected by centrifugation and lysed with buffer A (40 mM Tris-HCl pH7.4, 500 mM NaCl, 10% glycerol). The bacteria were lysed by sonication and the supernatant was flowed through a column packed with Ni-NTA. Proteins were eluted with buffer B (40 mM Tris-HCl pH7.4, 500 mM NaCl, 500 mM Imidazole) and purified with a Superdex 200 increase 10/300 column. Proteins were stored in buffer C (40 mM Tris-HCl pH7.4, 500 mM NaCl, 1 mM DTT) at -80°C.

For the phase separation experiments *in vitro*, MBP-EH1(FL)-GFP, MBP-EH1(ΔIDR3)-GFP and MBP-EH1(ΔCCΔIDR3)-GFP was cleaved with TEV protease for 3 hours to remove the MBP tag. Proteins were diluted to the desired concentrations in different concentration of NaCl. Droplets were observed in 384-well plate with a Zeiss LSM880 confocal microscope using a 63x oil 1.3 NA objective. For the *in vitro* partitioning assay, 5 µM EH1(FL)-GFP after MBP cleaved and 5 µM mCherry-TOL6 were mixed with buffer (40 mM Tris-HCl pH7.4, 100 mM NaCl) and incubated on ice for 30 min. Droplets were observed in a 384-well plate as above.

### TurboID proximity labelling

PSB-D *A. thaliana* cell suspension cultures were transformed with the AtEH1-linker-TurboID and experiments were performed as previously described^41^. The TPLATE-linker-TurboID dataset was described before^41^, and was re-analysed using the Mascot search engine. The datasets were compared to a large set of in-house TurboID experiments with unrelated baits performed using the same conditions. Proteins identified in at least two experiments, and showing a high (at least 20-fold) enrichment score (NSAF ratio × −log(P value)) versus the large dataset were considered enriched, similar as previously described^69^.

### Bioinformatics

All sequences used can be found in the Source Data file (tab Extended Data 2). For sequence analysis of individual proteins; prion-like residues were obtained using the PLAAC online tool with a core length of 60 and ‘relative weighting of background probabilities’ of 100. Prediction of disordered residues (MobiDB consensus scores) were obtained for the *A. thaliana* proteome (NCBI taxon ID 3702) on 2021-08-31. *A. thaliana* prion-like sequences were obtained from previous work^70,71^. For the classification of endocytic proteins (Fig. 4b); proteins were considered disordered if they had a MobiDB consensus score above 0.15 (15% disordered residues), and prion-like if they contained a prion-domain (≥ 60 amino acids). NARDINI+ analysis^36,37^ was performed as described using localCIDER^36,38^ to extract the sequences features. To normalize the compositional feature values, Z-scores were calculated from the dataset of the sequences used. Z-score vectors were hierarchically clustered using the Euclidian distance and complete linkage method.

### CLEM-ET

The CLEM approach was performed similarly to previously described with minor modifications^72^. For the CLEM-ET of AtEH1 condensates, a small region of the lower hypocotyl was cut from 4-day old *A. thaliana* seedlings stably expressing 35S:AtEH1-GFP. For the rapamycin experiments, seedlings were first treated with 2.5 µM rapamycin for 2 hours in 6 well plates with gentle shaking, and then a small region of the root tip was excised. Root or hypocotyl samples were quickly frozen in a Leica EM-PACT high-pressure freezer using 20% BSA as a cryoprotectant. Freeze-substitution was performed with a Leica AFS 2 in acetone containing 0.1% uranyl acetate. Samples were embedded in HM20 Lowicryl resin. 150nm ultra-thin sections were cut using a Leica Ultracut microtome with a diamond knife and placed on copper mesh grids.

EM grids were imaged before ET by fluorescence microscopy using a Zeiss LSM 880 confocal microscope with a 63x Oil NA 1.4 Apo objective. Transmission electron microscopy observations were carried out on a FEI TECNAI Spirit 120 kV electron microscope equipped with an Eagle 4Kx4K CCD camera. Correlation between fluorescence and electron microscope images was performed using the ec-CLEM plugin in Icy software. For hypocotyl samples, chloroplasts and vacuoles were used as landmarks for registration. For the root samples, cell edges were used as landmarks.

Electron tomography was performed as previously described^73^. Briefly, 5 nm gold beads were briefly applied to grids on both sides and used as fiducials for tomography. Tilt series were acquired from -65° to 65° at 1-degree increments. Pixel size was 0.28 nm or 0.39 nm at 4096x4096 for AtEH1-GFP condensates and rapamycin experiments, respectively. To acquire dual axis tomograms the grid was rotated 90°. The raw tilt series were aligned and reconstructed using the fiducial alignment mode with the eTomo graphical user interface in the IMOD software package. 10 to 25 fiducials were selected to accurately align images. Reconstruction was performed using the back-projection with SIRT-like filter (10-50 iterations) in IMOD.

### Segmentation and visualisation of EM tomograms

Ribosomes were segmented from tomograms using a pixel and object classification workflow in Ilastik. Ribosomes were manually annotated in 3D using the pixel classification method. The resulting probability map was smoothed in 3D (σ=3.5). Incorrectly annotated ribosomes were removed using a size filter, and object classification. Ribosome object predictions were imported into Amira as 8-bit labels together with ET tomography files. Mitochondria and clathrin-coated vesicles were manually segmented in Amira.

### Confocal microscopy

Unless otherwise indicated, all images were acquired using a PerkinElmer Ultraview spinning-disk system, attached to a Nikon Ti inverted microscope, and operated using Volocity software. Images were acquired on an ImagEM CCD camera (Hamamatsu C9100-13) using frame-sequential imaging with a 60x W NA 1.2 objective. Specific excitation and emission were performed using a 488 nm laser combined with a single band pass filter (500-550 nm) for GFP in single camera mode. RFP was visualized using 561 nm laser excitation and a 570-625 nm band pass filter. Exposure time was between 25-500 ms.

The time-lapse imaging of the rapamycin treated samples (Video S3) were performed using a microfluidics chip on a vertically mounted spinning-disk setup.

### Whole-mount immunofluorescence imaging

Roots from 4-day-old Arabidopsis seedlings were analysed by immunofluorescence as previously described^74^ using an InsituPro Vsi II (Intavis) automated system. Antibody dilutions were used as follows: rabbit anti-AtEH1 [1:600], rabbit anti-CHC [1:300], mouse anti-RFP [1:300], Alexa488-conjugated anti-rabbit IgG [1:600], Cy3-conjugated anti-mouse IgG [1:600].

### FM4-64 uptake imaging and analysis

Whole 5-day-old seedlings were incubated with 2 µM FM4-64 (Invitrogen) solution in half-strength MS liquid medium without sucrose at room temperature for 15 min. Samples were briefly washed twice with ½ MS before imaging. Images were acquired using the Leica SP8X system using a 40x W NA 1.2 objective using 488 nm laser excitation, and collection from 650nm - 750nm. The ratio of plasma membrane signal and cytosolic signal were calculated using an automated detection macro in ImageJ ‘detect_membrane_and_cytoplasm_from_a_roi.ijm’^16^, using a threshold set to 60.

### Fluorescence recovery after photobleaching (FRAP)

Tobacco cells transiently expressing proteins were incubated with Latrunculin B (4 μM) for 30 minutes prior to imaging to inhibit condensate movement. FRAP was performed on the spinning disk system using the Ultraview PhotoKinesis unit. Bleaching was performed using a 6×6 µm ROI using 100% of the 488 laser for 3s, images were acquired every 0.6 seconds for 120 seconds after bleaching. Fluorescence intensity was measured in Trackmate from tracked condensates. Analysis was performed using the easyFRAP online web tool using full-scale normalisation, and double exponential equation for curve fitting. For the 2in1 ratiometric FRAP experiment, AtEH1 WT and mutant reporters were expressed from a single T-DNA. FRAP was performed as above except images were acquired sequentially every 1 second. Due to variations in absolute t_1/2_ values between tobacco cells the represented FRAP curves are from 8 measurements binned to a t_1/2_ time of 17.2. For the ES9 FRAP experiment 4-day old seedlings were incubated for 3 minutes in 10 µM ES9 to inhibit endocytosis^67^, and individual endocytic foci were bleached using a 2×2 µm ROI with 25 % of the 488 nm laser to achieve a 70-80 % reduction in fluorescence.

For the FRAP experiments *in vitro*, MBP-AtEH1(FL)-GFP was cleaved with TEV protease for 3 hours to remove the MBP tag. AtEH1-FL protein was diluted into 10 µM at 100 mM NaCl. After 30 min of incubation on ice, droplets were bleached with a 488-nm laser pulse at 100% intensity on a Zeiss LSM880 confocal microscope using a 63x Oil NA 1.2 objective. Fluorescence recovery was recorded every 2 s for 400 s after bleaching.

### Condensate tracking

Tracking of condensate fusion, FRAP and mobility (was performed using Trackmate with the DoG detection algorithm. For the FRAP assay, the ‘extract track stack’ feature in Trackmate was used to generate the individual images.

### Endocytosis imaging and quantification of lifetime

Endocytosis dynamics were imaged on the UltraView spinning-disk system using a Nikon Perfect Focus System (PFSIII) and a 100x oil NA 1.4 objective. Images of root epidermal cells of 4-day old seedlings expressing pAtEH1:AtEH1-GFP were acquired at 2 fps for 5 minutes. Cells with very high density or high background signal were excluded from analysis as the signal-to-noise ratio was too low for automated quantitative analysis. Images were combined to 1 fps, and analysed in Matlab using a modified cmeAnalysis script^75^. Tracking parameters (cmeAnalysisTrackingAndDetection) were modified from default parameters as follows to improve tracking accuracy: TrackingGapLength [1], TrackingRadius [1 2]. Images of AtEH1 and DRP1a were acquired in dual-camera mode at 1 fps. Fluorescence intensity was measured using the ‘measure line profiles’ tool in the Volocity software. The start and end of the tracks were determined by measuring the signal intensity and comparing it to the background level.

### Surface rendering of condensates

Time-lapse images of AtEH1 IDR mutant condensates in *N. benthamiana* were acquired with the spinning disk system using a 60x objective and 1.5x optical zoom with scan settings set at ‘High Speed’. Z-stacks were acquired using 15 slices (0.5 µm spacing) at 2.54 seconds per stack, for a total duration of 4 minutes. Sample drift was corrected in 3D using the ‘Correct 3D drift’ plugin in Fiji. Images were imported into Imaris, and condensates were segmented using the ‘spots’ tool, coloured by ‘sphericity’. Note that the condensates in *N. benthamiana* have reduced sphericity in 3D due to being pressed between the plasma membrane and the vacuole (which occupies the majority of the cell volume).

### b-isox enrichment

5-day old Col-0 seedlings were ground in liquid nitrogen, and total proteins extraction and subsequent b-isox enrichment were performed as previously described^51^.

### SDS–PAGE and immunoblotting

For pEH1-AtEH1-GFP samples: Samples with 1× Laemmli loading buffer (BioRad) and 1× NuPage reducing agent (Invitrogen) were heated for 10 min at 95 °C. Samples were loaded and separated on a 4–20% SDS–PAGE TGX gel (BioRad) and subsequently blotted on polyvinylidenedifluoride (PVDF; BioRad). Membranes were blocked overnight at 4C in 5% skimmed milk in PBS-T. Next, the blots were incubated with the primary antibodies (α-TPLATE^13^, 1:1000], α-AtEH1^16^, 1:1000], α-CHC [Agrisera, AS10690, 1:2000], α-Tubulin [Sigma-Aldrich, T5168, 1:5000] and secondary antibodies (α-rabbit-HRP [Cytiva, NA934, 1:10000], α-mouse-HRP [Cytiva, NA931, 1:10000]) in 3% skimmed milk in PBST for 1 h. HRP-conjugated antibodies were detected using western lighting plus-ECL reagent (PerkinElmer).

### Multiple sequence alignment and phylogenetic analysis

To identify AtEH1 homologues, predicted proteins of selected genomes from the Phytozome v13 database were searched using the BLASTP algorithm with Arabidopsis AtEH1 as an input sequence. Multiple sequence alignment was constructed with the MAFFT algorithm in the einsi mode. Phylogenetic analysis was carried out utilizing PhyML v3.0 with the smart model selection. The phylogenetic tree was visualized using iTOL v6.

### Statistics and reproducibility

Statistical analysis was performed using Graphpad Prism and Microsoft Excel. Data was tested for normality for analysis. Significance criterion was set at a p value of <0.05. No statistical methods were used to predetermine sample size, but our sample sizes are similar to previously reported studies. Experiments were not randomised. Data collection and analysis were not performed blind to the conditions of the experiments. For the imaging of endocytic foci from pAtEH1:AtEH1-GFP seedlings cells with very high foci density, or high background signal were excluded as the signal-to-noise ratio was too low for automated quantitative analysis.

## Extended Data

## Supplementary Material

Supplementary material

## Data Availability

The *A. thaliana* proteome (NCBI taxon ID 3702) was analysed for prediction of disordered residues using the MobiDB database. The Phytozome v13 database was used to identify AtEH1 homologues. The mass spectrometry data have been deposited to the ProteomeXchange Consortium via the PRIDE partner repository with the dataset identifier PXD045068. Source data are provided with this study. All other data supporting the findings of this study are available from the corresponding author on reasonable request.

## Code availability

Code used in the manuscript are available online: ImageJ script detect_membrane_and_cytoplasm_from_a_roi https://github.com/pegro-psb/Cyto-PM-signal-quantification; ImageJ script MitoTally https://github.com/winkler-joanna/MitTally_NucTally; PlantCMEanalysis https://doi.org/10.5281/zenodo.3888519
