# Supplementary material for: Biomolecular condensation orchestrates clathrin-mediated endocytosis in plants
Source: Nat Cell Biol. Author manuscript; Available in PMC 2024 Sep 1. (PMC7615741; doi:10.1038/s41556-024-01354-6)
Supplement: Supplementary material [file EMS194255-supplement-Supplementary_material.zip › for annick NCB data/SourceData_UnprocessedBlots.pdf]

Dragwidge et al., Unprocessed blots

Fig. 1g

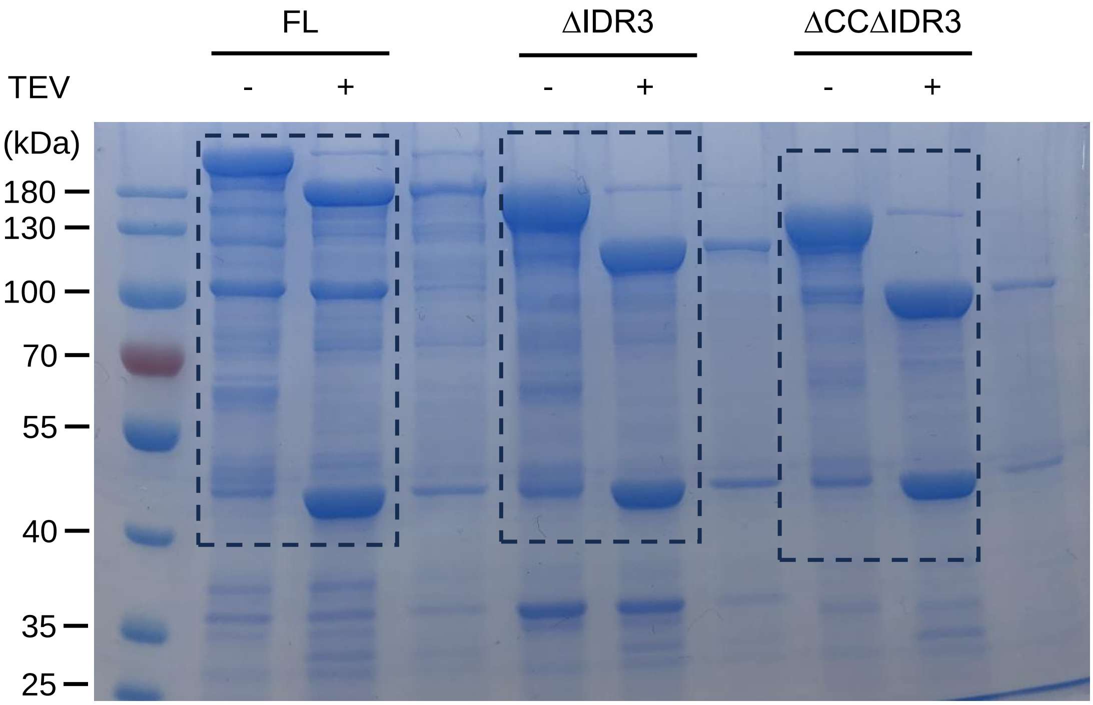

Fig. 6a: b-isox enrichment assay

Stainfree gel

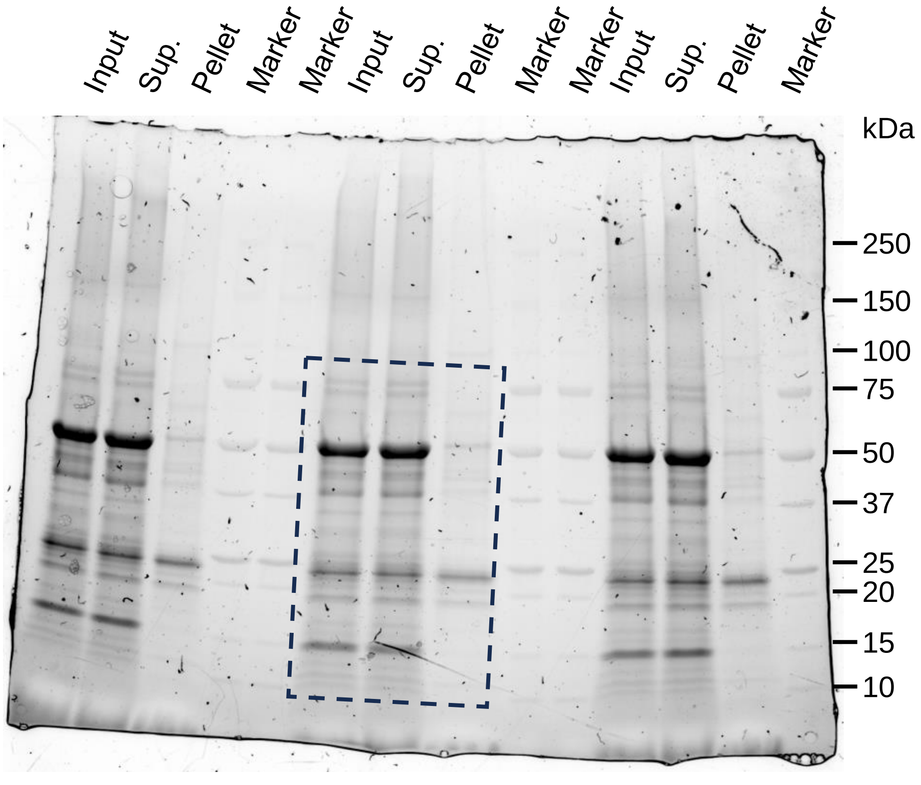

Fig. 6a: b-isox enrichment assay

αTPLATE

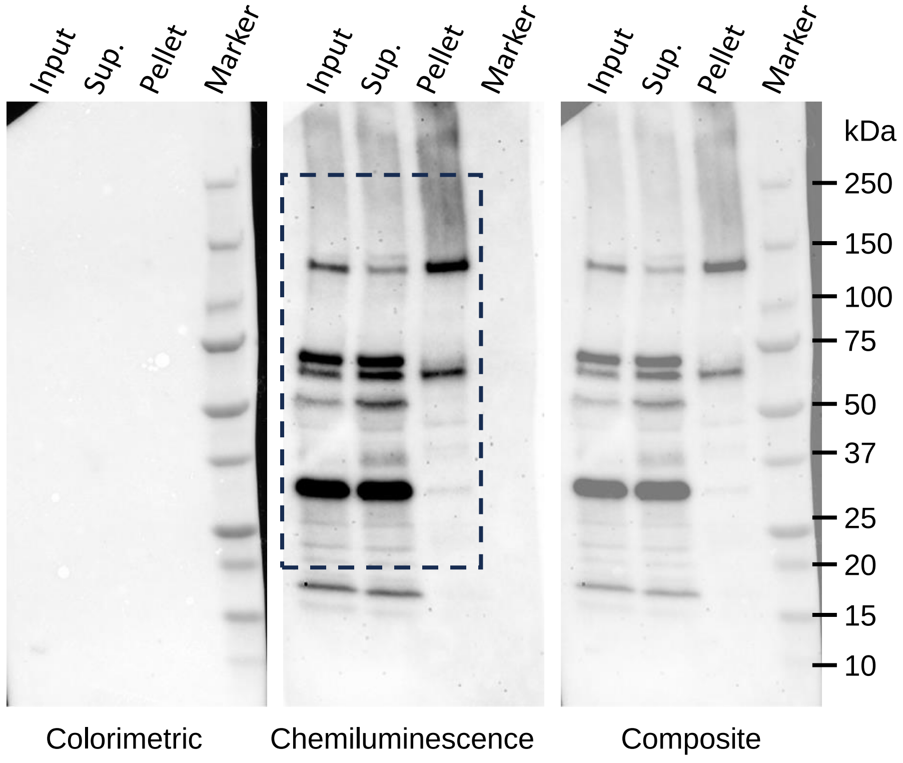

αEH1

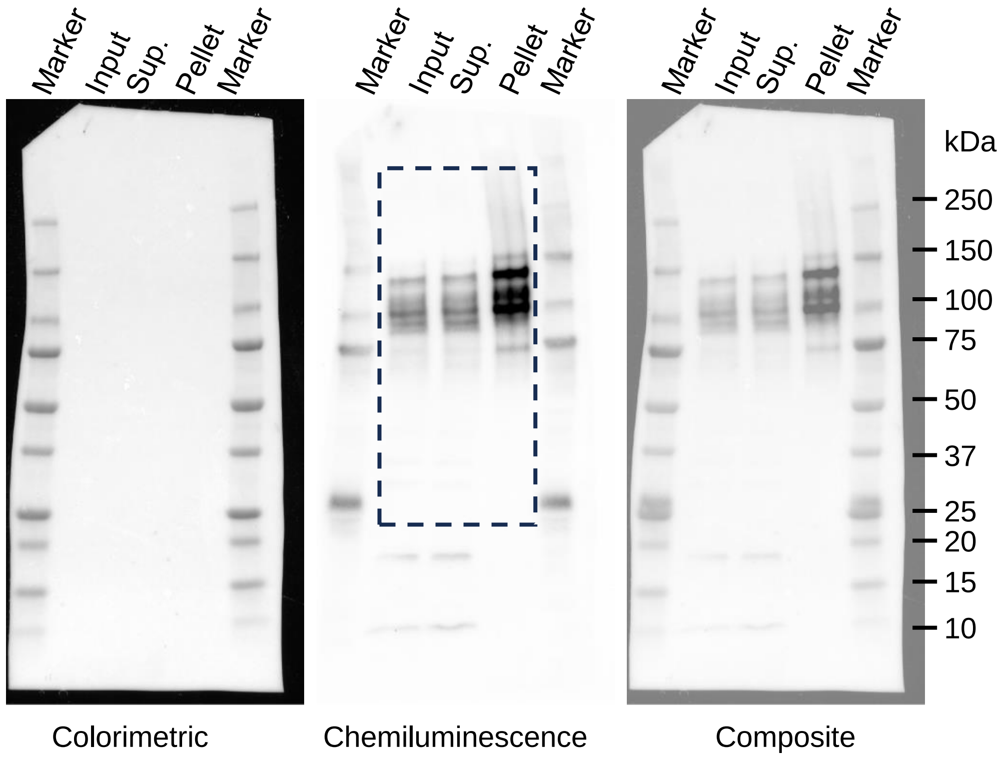

Fig. 6a: b-isox enrichment assay

αCHC

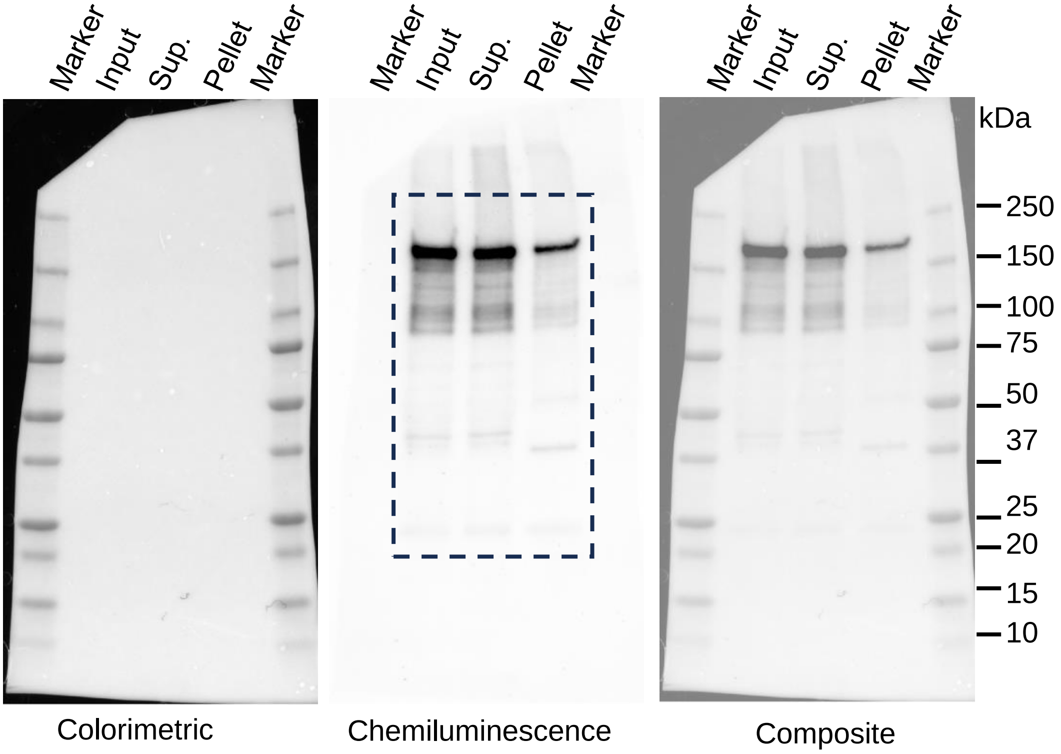

αTubulin

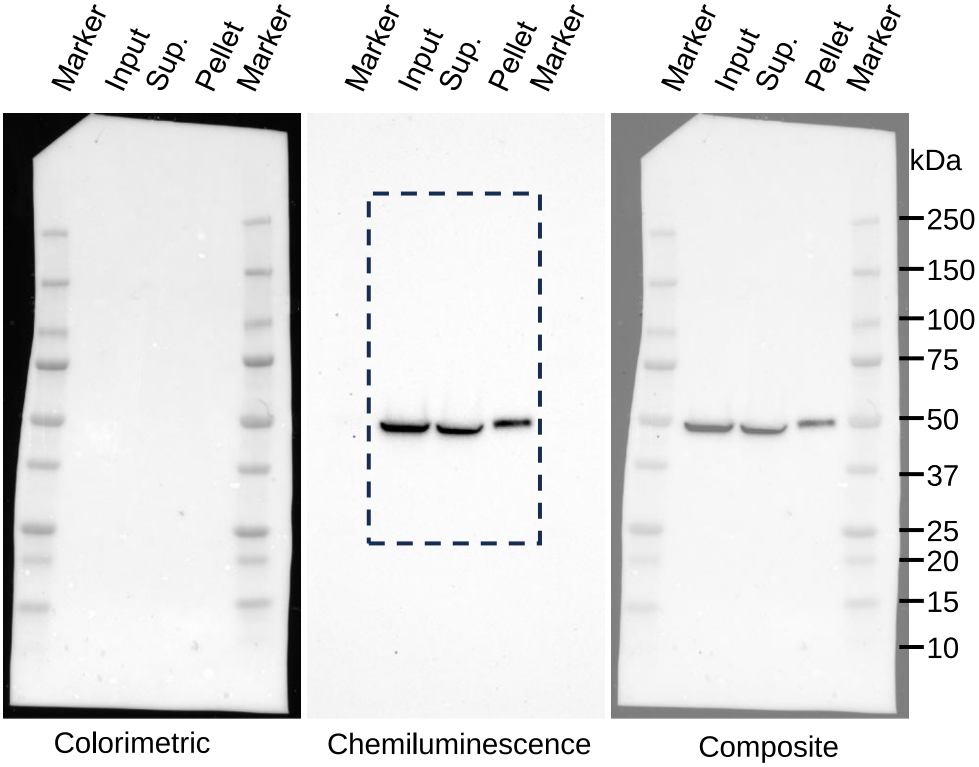

Dragwidge et al., Unprocessed blots

Fig. Extended Data 6b: Validation of Mito-TagBFP/TPLATE-mCh-FKBP *A. thaliana* lines

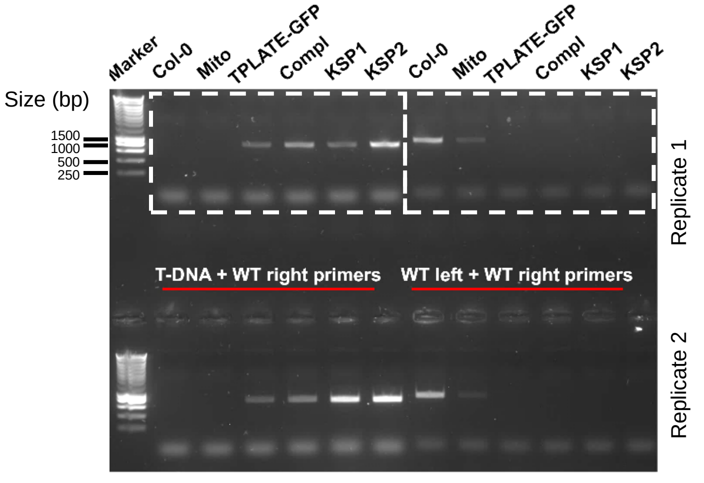

Fig. Extended Data 7b: b-isox enrichment assay

Stainfree gel

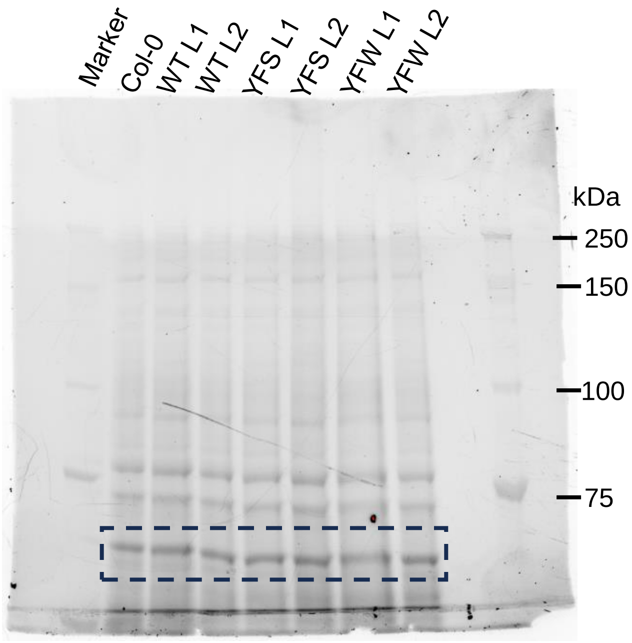

αEH1

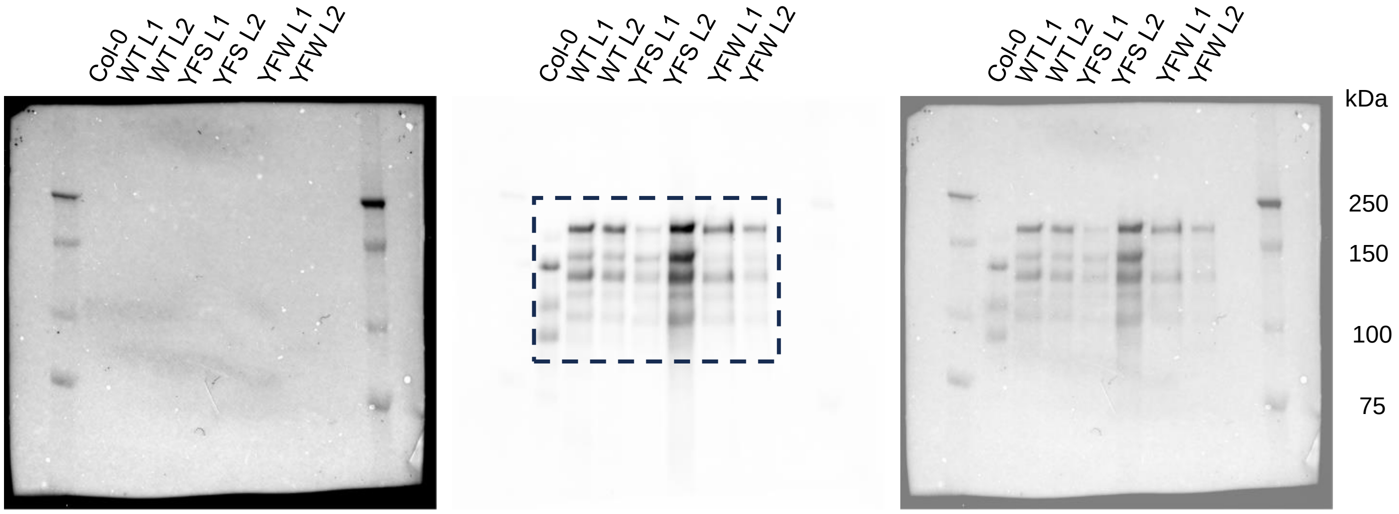

Colorimetric Chemiluminescence Composite
